# Structure–Emission Property Relationship of Bilayer 2D Hybrid Perovskites

**DOI:** 10.1021/jacs.5c04417

**Published:** 2025-04-30

**Authors:** Yumeng Song, Yifan Zhou, Congcong Chen, Kezhou Fan, Zhen Wang, Yu Guo, Ziming Chen, Lingling Mao, Jun Yin, Philip C. Y. Chow

**Affiliations:** † Department of Mechanical Engineering, 25809 The University of Hong Kong, Pokfulam 999077,Hong Kong, China; ‡ Department of Applied Physics, 26680 The Hong Kong Polytechnic University, Kowloon 999077, Hong Kong, China; § Department of Chemistry, 255310 Southern University of Science and Technology, Shenzhen, Guangdong 518055, China; ∥ Department of Physics, The Hong Kong University of Science and Technology, Clearwater Bay, Hong Kong 999077, China

## Abstract

Two-dimensional hybrid perovskites (2D-PVKs) have shown great promise for optoelectronic applications. However, the structure-emission property relationship of 2D-PVKs, particularly those with multiple octahedral layers in the metal-halide lattice (*n* > 1), is not fully understood. Here we combine experimental and theoretical studies to investigate a series of bilayer (*n* = 2) 2D-PVK crystals in both Ruddlesden–Popper (RP) and Dion–Jacobson (DJ) phases. Our results reveal that DJ-phase crystals exhibit a higher degree of octahedral lattice distortion compared with RP-phase crystals, with this distortion scaling inversely with interlayer spacing. Such octahedral distortion leads to (1) lower formation energies for iodine vacancies that act as nonradiative recombination centers, thereby reducing light emission yields, and (2) local inversion asymmetry that impacts electronic band structure and light emission properties. Among all the studied crystals, the DJ-phase crystal based on 4-(aminomethyl)­piperidinium cations demonstrates the largest intra- and interoctahedral distortions, leading to inversion asymmetry that causes significant Rashba band splitting and circular-polarization dependent photoluminescence at room temperature. Our results provide insights into the development of 2D-PVKs for future optoelectronic/spintronic applications.

## Graphical abstract

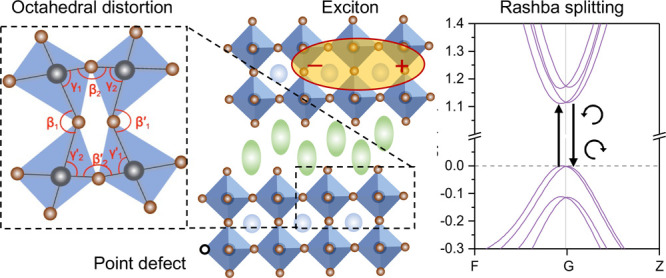

## Introduction

Hybrid organic–inorganic metal halide perovskites have emerged as a highly promising semiconductor material class suitable for a wide range of optoelectronic applications including solar cells,^1,2^ light-emitting diodes,^3,4^ X-ray scintillators,^5,6^ and photodetectors.^7,8^ Their advantages include simple synthesis, low-cost solution processing, large oscillator strengths, as well as tunable electronic properties and band structures. In particular, two-dimensional (2D) hybrid perovskites (2D-PVKs) have attracted much attention because they offer greater compositional/structural diversity and enhanced stability compared to their three-dimensional (3D) counterparts.^9,10^ The crystal structure of 2D-PVKs consists of metal-halide octahedral lattices, which are embedded with small organic cations sandwiched between layers of larger organic cations (spacers), forming a layered structure with alternating organic and metal-halide octahedral planes. The insulating organic spacers induce quantum and dielectric confinement effects, leading to the formation of bound electron–hole pairs (excitons) that can recombine radiatively in high yields. Furthermore, the distinct couplings between the soft metal-halide octahedral lattice and the band-edge excited states give rise to various phenomena, including exciton-polaron formation,^11,12^ coherent phonon interactions,^13^ spin-polarized light emission,^14^ and Rashba band splitting.^15,16^ These effects demonstrate promising implications for the advancement of future optoelectronic and spintronic devices.

2D-PVKs generally adopt either a Ruddlesden–Popper (RP) phase or a Dion–Jacobson (DJ) phase.^17,18^ In the RP phase, the metal-halide octahedral lattice is separated by double layers of alternating monoammonium cation spacers, while a single layer of diammonium cation spacer is present in the DJ phase. The general formula for RP and DJ phases is A′_2_A_*n*–1_B*_n_* X_3*n*+1_ and A″A_*n*–1_B*_n_* X_3*n*+1_, respectively, where A′ (monovalent) and A″ (divalent) are the larger organic spacer cations between adjacent metal-halide octahedral lattices; A is the small organic cation embedded within the metal-halide octahedral lattices; B is the metal cation (typically Pb or Sn) and X is the halide anion; *n* (≥1) is the number of octahedral layers in the metal-halide lattice, as shown in Figure 1a. Increasing the number of octahedral layers (*n* > 1) results in reduced exciton binding energies and red-shifted emission spectra.^19^

**Figure 1:**
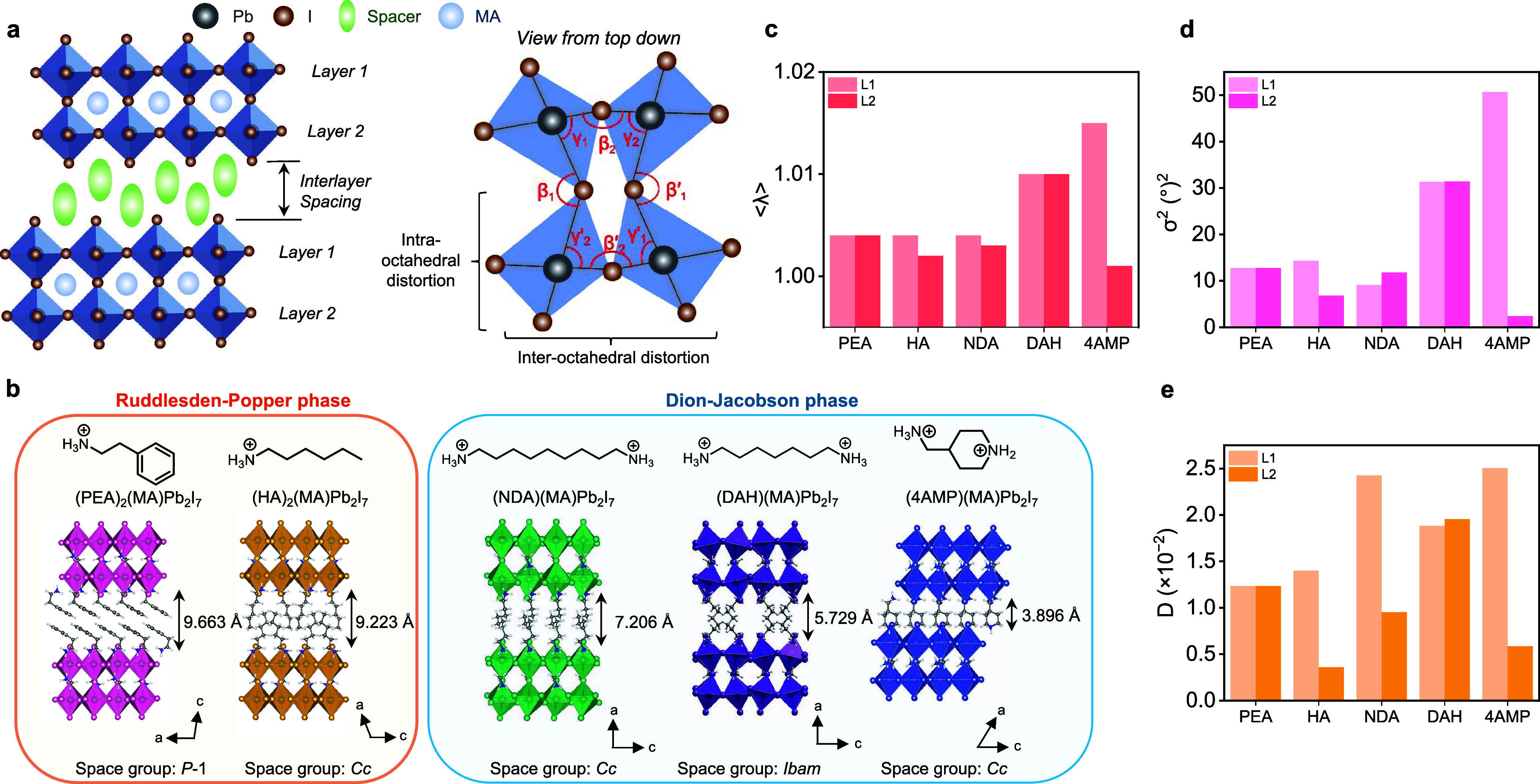
(a) Schematic illustration of the crystal structure of bilayer (*n* = 2) 2D-PVK. The equatorial Pb–I–Pb bond angles (β and β′) and I–Pb–I bond angles (γ and γ*′*) in the top (L1) and bottom (L2) octahedral layers can be used to quantify the inter- and intraoctahedral distortions, respectively. (b) Crystal structures of the 2D-PVK crystals involved in this study: (PEA)_2_(MA)­Pb_2_I_7_, (HA)_2_(MA)­Pb_2_I_7_, (NDA)­(MA)­Pb_2_I_7_, (DAH)­(MA)­Pb_2_I_7_, and (4AMP)­(MA)­Pb_2_I_7_. PEA and HA belong to RP-phase crystals, while NDA, DAH, and 4AMP belong to DJ-phase crystals. Comparison of the (c) quadratic elongation (⟨λ⟩), (d) bond angle variance (σ^2^), and (e) distortion index (*D*) of top/bottom octahedral layers for the studied bilayer 2D-PVKs.

The extensive library of organic spacer cations compatible with 2D-PVKs has enabled the development of numerous 2D-PVKs with both RP and DJ phases in recent years.^10,20^ The choice of the organic spacers plays a critical role in determining the structural, electronic, optical, and photophysical properties of 2D-PVKs. This has been extensively studied by Kanatzidis and co-workers, among other researchers, who have synthesized a wide range of RP- and DJ-phase 2D-PVKs utilizing various organic spacers.^19–27^ Other studies indicate that using rigid organic spacers in single-layer 2D-PVKs (*n* = 1) enhances exciton lifetimes and emission yields, and can accelerate strain propagation by influencing electron–phonon interactions.^28–30^ Additionally, varying the length and structural rigidity of spacer cations significantly affects the stability of both RP- and DJ-phase 2D-PVKs.^27,31,32^ Recent work by Guo et al. demonstrates that optimizing cation rigidity enhances 2D-PVK stability by inducing coadaptation between organic spacers and metal-halide octahedra.^33^

While there is a general consensus that lattice softness and crystal structure properties impact the physical properties of both RP- and DJ-phase 2D-PVKs, there is still limited knowledge of the underlying structure–emission property relationships, especially for systems with *n* > 1. Gaining a deeper understanding of this relationship will facilitate systematic design and synthesis of new organic spacers for high-performance 2D-PVK-based optoelectronic and spintronic devices.

## Results and Discussion

### Sample Preparation

Here we use a combination of experimental and theoretical methods to study the structure-emission relationship of bilayer (*n* = 2) 2D-PVK crystals in both RP- and DJ-phases. Our study involves five bilayer 2D-PVK single crystals, namely, (PEA)_2_(MA)­Pb_2_I_7_ (PEA = phenylethylammonium), (HA)_2_(MA)­Pb_2_I_7_ (HA = hexylammonium), (NDA)­(MA)­Pb_2_I_7_ (NDA = nonyldiammonium), (DAH)­(MA)­Pb_2_I_7_ (DAH = 1,7-heptanediamine) and (4AMP)­(MA)­Pb_2_I_7_ (4AMP = 4-(aminomethyl)­piperidinium). The chemical structures of these bilayer 2D-PVK crystals are shown in Figure 1b, and the details of the synthesis and experimental methods can be found in the Supporting Information. For clarity, we refer to these bilayer 2D-PVK crystals by their organic spacer abbreviations. The PEA and HA crystals belong to the RP-phase, while the NDA, DAH, and 4AMP crystals belong to the DJ-phase. We studied exfoliated crystal flakes either on precleaned silicon wafers or quartz substrates for optical experiments, with crystal thicknesses ranging from 250 to 450 nm (Figures S1 and S2).

### Crystal Structure Analysis

We first investigated the structural properties of bilayer 2D-PVK crystals with various organic spacers by performing geometry optimization of the crystal structures using density functional theory (DFT) calculations. For PEA, HA, NDA, and 4AMP, we optimized the crystal structures using crystallographic information files (CIF) available from the literature.^18,21,27,34^ For DAH, we acquired the CIF from single-crystal X-ray diffraction measurements (Table S1), as it has not been published to the best of our knowledge. We found good agreement between the experimental powder X-ray diffraction (PXRD) patterns and the simulated PXRD patterns of all crystal structures optimized by DFT, confirming the phase purity of our synthesized bilayer 2D-PVK crystals (Figure S3). All DFT-optimized geometric parameters of the various crystal structures are summarized in Figure S4 and Tables S2–S4.

As illustrated in Figure 1a, the metal-halide (Pb–I) octahedra in both top (L1) and bottom (L2) layers of the bilayer 2D-PVKs experience intraoctahedral and interoctahedral distortions, which can be identified in our DFT analysis. We then quantified both types of distortion in L1 and L2 for each 2D-PVK crystal, following the approach established in previous studies.^35–37^ First, intraoctahedral distortion can be quantified using descriptors of quadratic elongation (⟨λ⟩), bond angle variance (σ^2^), and distortion index (*D*), which follow eqs 1–3, respectively:

$$\langle \lambda \rangle =\sum_{i=1}^{6}{\left(\frac{l_{i}}{l_{0}}\right)}^{2}/6$$ 1

$$\sigma^{2}=\sum_{i=1}^{12}(\theta_{i}-90{)}^{2}/11$$ 2

$$D=\frac{1}{6}\sum_{i=1}^{6}\frac{\left|l_{i}-l_{\mathrm{av}}\right|}{l_{\mathrm{av}}}$$ 3

where *l*_*i*_, *l*_av_, and θ_*i*_ stand for each individual Pb–I bond length, average Pb–I bond length, and each I–Pb–I bond angle, within a single Pb–I octahedron, respectively; *l*_0_ represents the Pb–I bond length in a regular Pb–I octahedron of the same volume. We analyzed these structural parameters across eight Pb–I octahedra in the supercell representation of both top and bottom layers for each crystal (see Figure S4 for details), and the average values of ⟨λ⟩, σ^2^, and *D* are summarized in Figure 1c–e. Larger values of these parameters indicate a higher degree of intraoctahedral distortion.

The average ⟨λ⟩ and σ^2^ show that overall, the RP-phase crystals (PEA and HA, with interlayer spacings of 9.663 and 9.223 Å, respectively) have a lower degree of intraoctahedral distortion compared to the DJ-phase crystals (NDA, DAH, and 4AMP). Although NDA, which has the longest interlayer spacing (7.206 Å) among the studied DJ-phase crystals, shows similar ⟨λ⟩ and σ^2^ values as the two RP-phase crystals, its nearly 2-fold greater average *D* value in the top layer still indicates more severe intraoctahedral distortion. While DAH (interlayer spacing of 5.729 Å) shows relatively balanced intraoctahedral distortion metrics in the top/bottom layers, all descriptors are significantly larger than those in the RP-phase crystals, also indicating its larger level of intraoctahedral distortion. Interestingly, we find that although the bottom layer of 4AMP (interlayer spacing of 3.896 Å) shows a low degree of intraoctahedral distortion according to all descriptors, its top layer experiences the largest degree of intraoctahedral distortion among all the layers studied herein.

In addition, the degree of interoctahedral distortions can be quantified using the equatorial Pb–I–Pb angle β and its disparity Δβ (Δβ = β_1_ – β_1_′ or β_2_ – β_2_′), as demonstrated in Figure 1a.^36,37^ Values of β approaching 180° and Δβ approaching zero would indicate a low degree of interoctahedral distortion. Both the top and bottom layers in the two RP-phase crystals show β ranging between ∼150°–157° and a Δβ of less than 3.3°, thus indicating a relatively low degree of interoctahedral distortion (full data listed in Table S2). Considerably greater degrees of interoctahedral distortion are observed in the DJ-phase crystals, evidenced by the large maximum Δβ ranging from 13° to 27°. 4AMP and DAH emerge as the crystals with the largest degree of interoctahedral distortion, with the smallest β (∼142° for both) and maximum Δβ of ∼18° and ∼27°, respectively. More specifically, our results show that the top layer (L1) of 4AMP suffers from significant intra- and interoctahedral distortions, while its bottom layer (L2) is composed of relatively nondistorted Pb–I octahedra. Low-frequency Raman spectroscopy data (Figure S5) show sharper scattering peaks for the RP-phase crystals, with increasingly broad features observed for the DJ-phase crystals with decreasing interlayer spacing, indicating a more distorted lattice with higher anharmonicity.^38–40^

Next, we investigated the structural centrosymmetry of the bilayer 2D-PVK crystals, which is known to be closely linked to spin-dependent phenomena such as Rashba band splitting. As established by Blum, Mitzi and co-workers for monolayer (*n* = 1) 2D-PVK crystals,^36,41^ the local inversion asymmetry in the Pb–I octahedral layer can be quantified by the in-plane projected Pb–I–Pb and I–Pb–I bond angle differences (Δβ_in_ and Δγ_in_, respectively), where Δβ_in_ = β_1‑in_ – β_1‑in_′ or β_2‑in_ – β_2‑in_′ (β_1‑in_, β_1‑in_′, β_2‑in_, and β_2‑in_′ represent the corresponding in-plane projected angles of β_1_, β_1_′, β_2_ and β_2_′, respectively, and analogical definition for the Δγ_in_), as demonstrated in Figure 2a. Figure 2b,c show the maximum Δβ_in_ and Δγ_in_ for both top/bottom octahedral layers for the various bilayer 2D-PVK crystals. Notably, the top layer in the 4AMP crystal shows significantly larger maximum Δβ_in_ and Δγ_in_ (both ∼18°) compared to those found in the other crystals (below ∼7°), thus indicating that this system has the largest local inversion asymmetry among all five crystals. We will return to this topic below.

**Figure 2:**
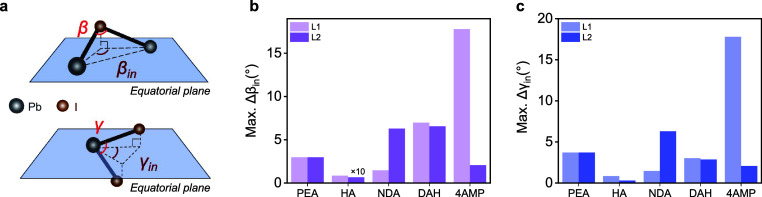
(a) Schematic diagram showing the space angle of β (Pb–I–Pb) and γ (I–Pb–I) within the Pb–I octahedron, as well as their projected angles into the equatorial plane (β_in_ and γ_in_, respectively). The local inversion asymmetry in the Pb–I octahedral layer can be quantified by the maximum in-plane projected Pb–I–Pb and I–Pb–I bond angle differences (Max. Δβ_in_ and Max. Δγ_in_, respectively), where the definitions of Δβ_in_ and Δγ_in_ are described in the main text. Summary of the (b) Max. Δβ_in_ and (c) Max. Δγ_in_ angles for the top (L1) and bottom (L2) octahedral layers in all five bilayer 2D-PVK crystals.

### Light Emission Properties

Despite the differences in structural properties, all five bilayer (*n* = 2) 2D-PVK crystals show similar optical absorption and photoluminescence (PL) spectra (Figure 3a). At room temperature, a clear excitonic resonance absorption feature and PL emission peak at approximately 580 nm are observed for all samples, indicating their exciton-dominant nature and comparable optical bandgaps. We compared the PL intensities of these bilayer 2D-PVK crystals across a range of excitation fluences (Figure 3b). A significant variation in PL intensity, spanning up to 2 orders of magnitude, was observed, with the intensity decreasing in the order: PEA > HA > NDA > DAH > 4AMP crystals (Figures S6). Fitting the fluence-dependent PL with a power law revealed *b* values slightly above 1, ranging between 1.1 and 1.4. This suggests that although the bilayer 2D-PVK crystals mainly retain excitonic characteristics, a degree of nonexcitonic behavior is also present, likely due to the reduced exciton binding energy compared to their monolayer (*n* = 1) counterparts that have a strong confinement nature.

**Figure 3:**
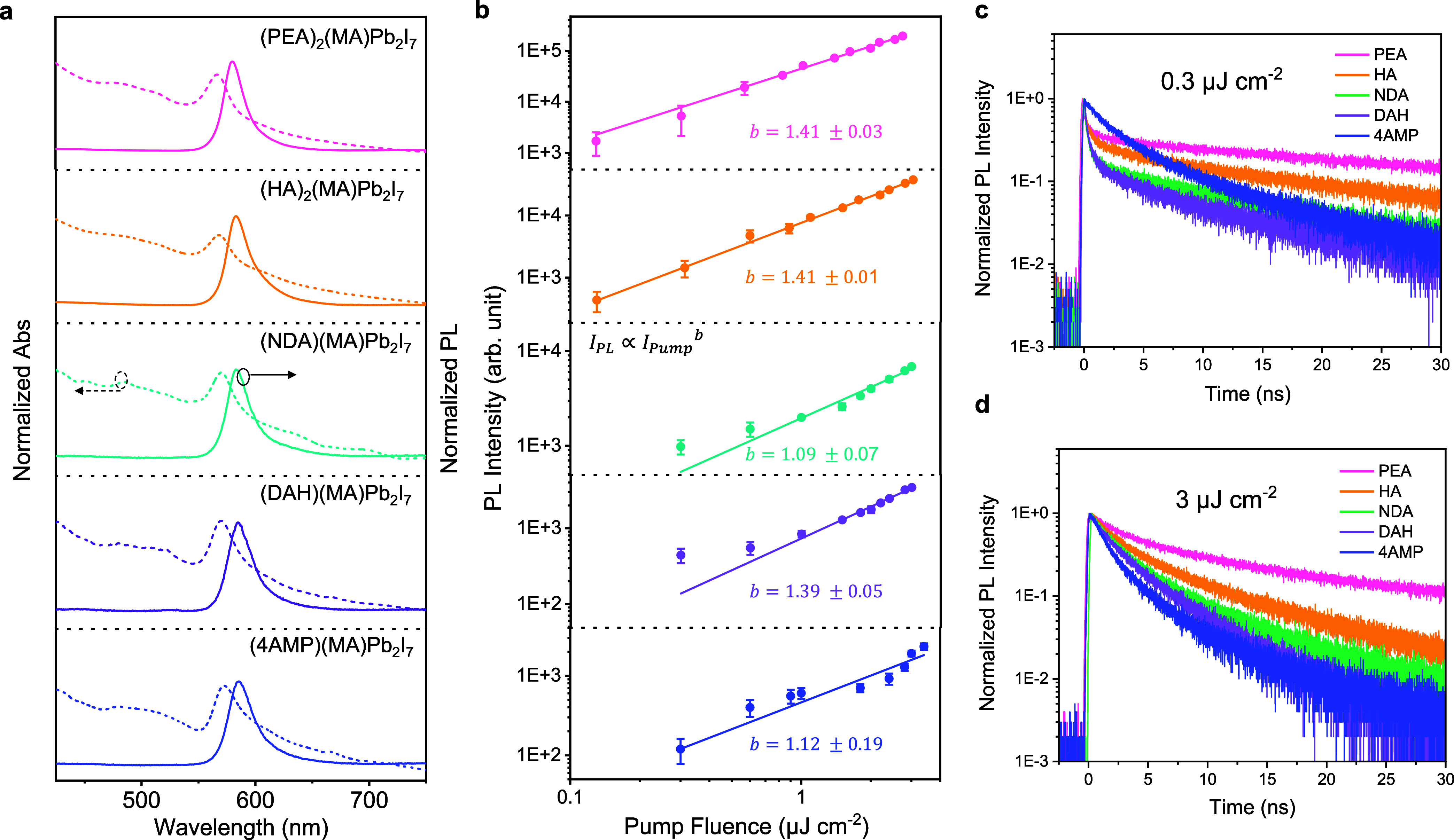
(a) Normalized absorbance spectra and PL spectra under 520 nm excitation at room temperature. (b) PL peak (580 nm) intensity as a function of pump fluence. Solid lines represent power law fits, where *I*_PL_ and *I*_Pump_ represent the PL intensity and pump fluence, respectively. TRPL decay kinetics under the pump fluences of (c) 0.3 μJ cm^–2^ and (d) 3 μJ cm^–2^, excited at 520 nm and probed at 580 nm.

We subsequently performed time-resolved photoluminescence (TRPL) decay measurements on these crystals at a low excitation fluence of 0.3 μJ cm^–2^, corresponding to an initial exciton/carrier concentration of 5.6 × 10^15^ cm^–3^, which places the bilayer 2D-PVK in the first-order recombination regime (see Supporting Information for details).^3,4^ As shown in Figure 3c, the PEA, HA, NDA, and DAH crystals all exhibit biexponential decay kinetics, consisting of a fast decay component (approaching the time resolution of our instrument) and a slow decay component (lasting tens of nanoseconds). The fast decay component results from the rapid depopulation of excitons/carriers, likely due to trap filling processes as reported previously.^42,43^

To further investigate this, we increased the pump fluence from 0.3 μJ cm^–2^ to 3 μJ cm^–2^ (corresponding to an exciton/carrier concentration of 5.6 × 10^16^ cm^–3^). As shown in Figures 3d and S7a–d, the fast decay component gradually diminishes, indicating increasing trap saturation at higher fluences, which reduces the contribution of trap filling to overall exciton/carrier kinetics. The slow decay component, which reflects the dynamics of rest band-edge excitons and carriers, exhibits relatively similar decay rates at low fluences. However, at high fluence, the decay accelerates, indicating the onset of second-order processes, such as exciton–exciton annihilation or free electron–hole recombination, in these quasi-excitonic systems. Therefore, to precisely extract the trap filling time, we used biexponential decay to globally fit the low-fluence kinetics to eliminate the impact of higher-order processes (Figure S8). In this scenario, we observed a decrease in trap filling time (τ_1_) from 0.36 ns for the PEA sample to 0.34 ns (HA), 0.27 ns (NDA), and 0.26 ns (DAH). The shorter trap filling times likely result from a higher density of traps in the perovskites, which accelerates exciton/carrier trapping. Consequently, our results suggest that the overall trap density and associated trap-assisted nonradiative recombination increase in the order: PEA < HA < NDA < DAH, consistent with the observed trend in PL intensity. This trend in trap density (i.e., PEA < HA < NDA < DAH) is further supported by the increasing proportion of the trap filling process (τ_1_) in the overall exciton/carrier kinetics, as shown in Figure 3c.

However, the behavior of the 4AMP crystal deviates significantly from that of the aforementioned crystals. In the fluence-dependent measurements, as shown in Figure S7e, the fast trap-filling process appears to be absent, with only second-order recombination of excitons/carriers observed. Two plausible yet contrasting explanations could account for this change in TRPL decay dynamics: (i) the trap density in the 4AMP crystal is sufficiently low, such that the lowest pump fluence of 0.3 μJ cm^–2^ is already high enough to saturate all trap states, or (ii) the 4AMP crystal exhibits an exceptionally high trap density, leading to an ultrafast trap-filling process that exceeds the temporal resolution of our TRPL setup (∼0.2 ns). Given the inferior PL properties of the 4AMP sample compared to other crystals, the latter scenario appears more likely, although further experimental evidence is required to confirm this hypothesis.

### Point Defect Formation Energies

To understand the variations in emission and nonradiative recombination properties between these bilayer 2D-PVK crystals, we calculated the formation energies of various types of point defects based on the aforementioned DFT-optimized crystal structures, namely: (i) organic spacer vacancies (V_Spacer_), (ii) in-plane iodine vacancies (V_I‑in_), and (iii) out-of-plane iodine vacancies (V_I‑out_). Figure 4a shows the schematic illustration of these point defects, and the corresponding defect formation energies for the bilayer 2D-PVK crystals are shown in Figure 4b–d. We determined the feasible chemical potential regions for the thermal equilibrium growth of these bilayer 2D-PVK crystals at the GGA/PBE+vdW level (Figure S9) and calculated the formation energies of the considered point defects under three different growth conditions (i.e., Pb-poor/I-rich, moderate, and Pb-rich/I-poor; see Figure S10 for details).

**Figure 4:**
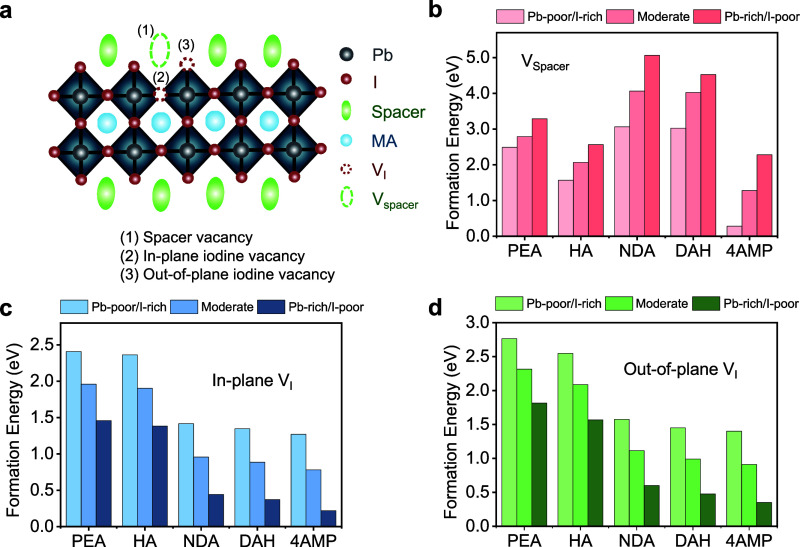
(a) Schematic illustration of bilayer 2D-PVK crystal lattice with various point defects, namely: (i) organic spacer vacancies (V_Spacer_), (ii) in-plane iodine vacancies (V_I‑in_), and (iii) out-of-plane iodine vacancies (V_I‑out_). Calculated formation energies for various point defects of (b) V_Spacer_, (c) V_I‑in_, and (d) V_I‑out_ in the bilayer 2D-PVK crystals at three different growth conditions (i.e., Pb-poor/I-rich, moderate, and Pb-rich/I-poor), as calculated at the GGA/PBE+vdW level.

Our results show that the formation energies of iodine vacancies (V_I_) in the two RP crystals (PEA and HA) are significantly higher than those in the three DJ crystals (NDA, DAH, and 4AMP). Specifically, the formation energies of V_I‑in_ and V_I‑out_ scale in the order of PEA > HA > NDA > DAH > 4AMP, following the same trend as for the emission intensity. Note that there is also a general decrease in the formation energy of V_I_ when shifting from Pb-poor/I-rich to Pb-rich/I-poor conditions. The low V_I‑in_ and V_I‑out_ formation energies for the NDA, DAH, and 4AMP are likely to result in a high density of point defect sites in the perovskite octahedral lattice, which can act as nonradiative recombination centers for the excitons/carriers, which mainly reside along the octahedral lattice. While we also find higher V_Spacer_ formation energies in PEA and HA crystals compared to 4AMP (Figure 4b), our result shows that the NDA and DAH crystals have the highest V_Spacer_ formation energy among these five crystals. We therefore consider that the presence of iodine vacancies in the octahedral lattice (both in-plane and out-of-plane) is more directly related to the emission intensity and trap filling rates in these bilayer 2D-PVK crystals compared to organic spacer vacancies. Based on our crystal structure analysis above, we believe that the significantly lower formation energies of iodine vacancies in the DJ-phase crystals compared to RP-phase crystals are inherently related to the larger intra- and interoctahedral distortions within the lattice that lead to nonuniform bond angles and lengths, especially in the top octahedral layer (L1).

### Rashba Band Splitting

As mentioned above (Figure 2), 4AMP crystal exhibits the largest local inversion asymmetry among the five bilayer 2D-PVK crystals as characterized by the high in-plane projected Pb–I–Pb and I–Pb–I bond angle differences,^36,41^ specifically in its top layer. This is supported by the observation of second harmonic generation (SHG) in the 4AMP crystal (Figure S11). We proceeded to perform DFT calculations on the electronic band structures of these bilayer 2D-PVK crystals (Figure 5a). It is noteworthy that the discrepancy between calculated and experimental bandgaps determined by absorption/emission measurements (Figure 3a) can be attributed to the underestimation of bandgaps introduced by the inclusion of spin–orbit coupling (SOC) effects in our calculations.^44,45^ In agreement with the structural analysis, we observed clear Rashba band splitting for the 4AMP crystal, characterized by a splitting energy of *E*_R_ = 4.782 meV and Rashba coefficient of α = 0.496 eV/Å. A slight band splitting is found for the NDA crystal (*E*_R_ = 0.841 meV, α = 0.045 eV/Å), and no notable band splitting is observed for PEA, HA, and DAH crystals. We note that past studies have reported HA crystal with both *C*2/*c* (centrosymmetric) and *Cc* (noncentrosymmetric) space groups,^26,27^ and we confirm that no clear signs of Rashba splitting in the electronic band structure are found in either case (Figure S12). Our results are also consistent with previous works on monolayer (*n* = 1) 2D-PVK crystals that the intra- and interoctahedral distortions provide a more reliable descriptor of local inversion symmetry breaking that induces Rashba splitting in 2D-PVKs compared to the global space group^27,36,41^ [e.g., both HA and NDA also have noncentrosymmetric space group (*Cc*) but do not exhibit distinguishable Rashba splitting].

**Figure 5:**
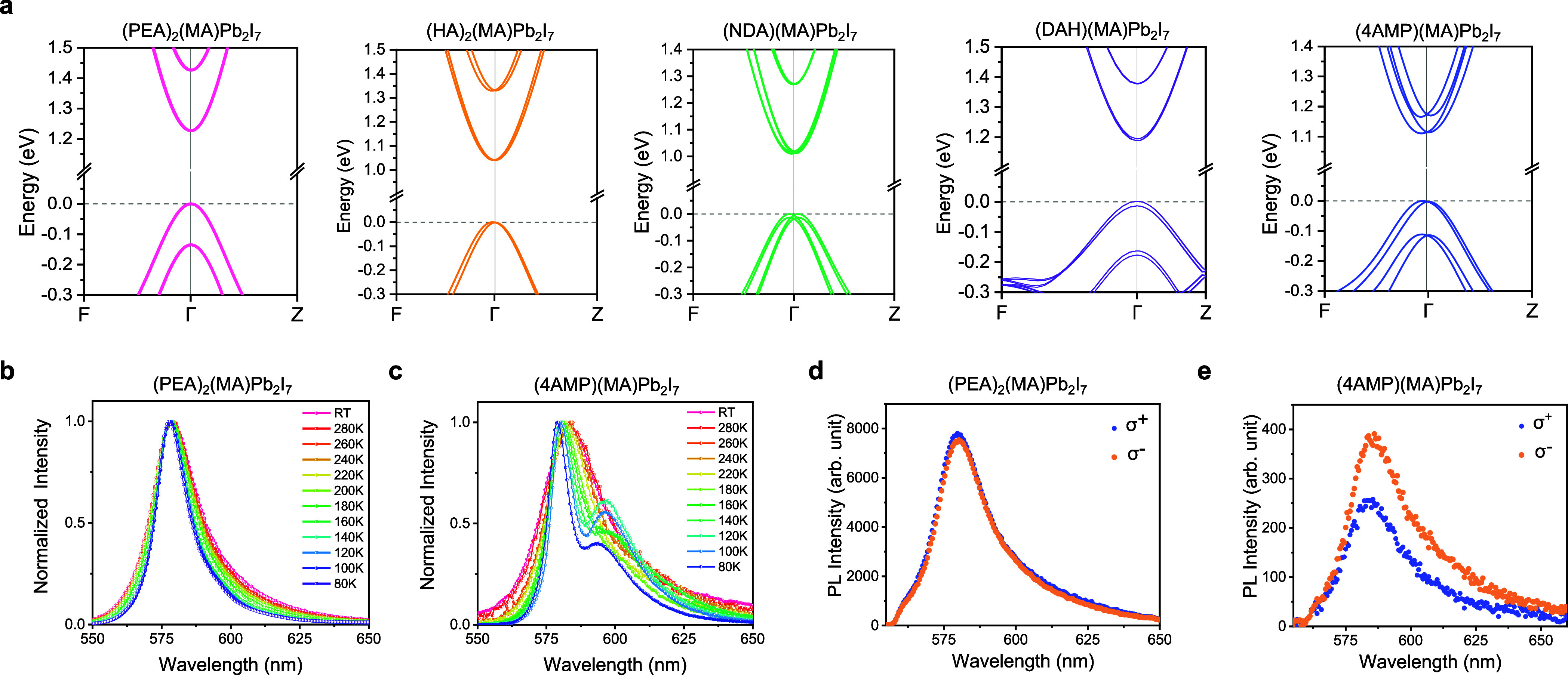
(a) Electronic band structures of various bilayer 2D-PVK crystals as calculated at the GGA/PBE+vdW+SOC level using DFT theory. Temperature-dependent PL spectra of (b) PEA and (c) 4AMP crystals under 520 nm laser excitation. Left (σ−) and right-handed (σ+) circularly polarized PL of (d) PEA and (e) 4AMP crystals under right-handed (σ+) circularly polarized excitation at 520 nm. Corresponding data for HA, NDA, and DAH crystals can be found in Figure S15.

In low temperature PL measurements, besides observing a significant increase in emission intensity (associated with reduced exciton/carrier nonradiative recombination) and spectral narrowing (associated with reduced electron–phonon couplings), we observed a clear splitting of the emission spectrum for the 4AMP crystal as the temperature dropped below ∼180 K (Figure 5c). At 100 K, two emission peaks centered at ∼580 and ∼596 nm are observed (Figure S13). Tracking of the TRPL decay kinetics at these emission energies reveals different decay rates, indicating that these two emission features originate from different electronic transitions. Since no splitting in the emission spectrum is observed for the other four crystals with inversion symmetry at low temperatures (Figure 5b for PEA and Figure S14 for HA, NDA, and DAH crystals), we consider that it arises from the Rashba band splitting in the asymmetric 4AMP crystal. We note that, for the HA crystal, a broad emission feature between ∼650 and ∼750 nm emerged at below ∼180 K (Figure S14b). We tentatively assign this to the emission of self-trapped excitons, which has been reported for various 2D-PVKs at low temperature.^46,47^ Circular polarization-resolved photoluminescence (CPL) measurements were performed at room temperature to further investigate the role of Rashba band splitting in emission properties. The presence of Rashba splitting gives rise to spin-split bands with opposite optical helicity; thus, under excitation by light of a particular polarization, the intensity of left (σ^–^) and right (σ^+^) circularly polarized PL is expected to be different.^16,48^ Such a result is indeed observed for the 4AMP crystal (Figure 5e). On the other hand, similar left and right circularly polarized PL emission features were observed for the other four crystals, which is consistent with the lack of a significant Rashba band splitting (Figure 5d for PEA and Figure S15 for HA, NDA, and DAH crystals).

Finally, while our results demonstrate that the selected bilayer 2D-PVK crystals with the RP-phase generally show reduced octahedral distortions and higher iodine vacancy defect formation energies than their DJ-phase counterparts, the organic spacer cations in the selected RP/DJ-phase crystals have different chemical structures and functional groups. Systematic study and comparison between RP/DJ-phase crystals based on spacers with highly similar chemical structures and functional groups are worth exploring in future studies. To this end, we have attempted to synthesize and study DJ-phase bilayer 2D-PVK based on 2-[4-(2-aminoethyl)­phenyl]­ethanamine (APEA) and hexane-1,6-diamine (HDA) cations, which share structural similarities with the phenylethylammonium (PEA) and hexylammonium (HA) monoammonium cations in the aforementioned RP-phase crystals. However, these two crystals were difficult to obtain in the pure *n* = 2 phase, preventing us from conducting reliable experimental characterizations. Nevertheless, we conducted DFT calculations to theoretically study both the lattice distortion and defect formation energies of both APEA and HDA *n* = 2 crystals (Figure S16), and the results are largely consistent with our findings for the other DJ-phase crystals described above. Also, regarding our TRPL analysis, we note that a number of studies have revealed exciton-polaron formation and dynamic disorder-induced charge transfer state formation in 2D-PVK crystals.^49,50^ Differences in these properties among the bilayer 2D-PVK crystals studied herein may also lead to changes in the dipole moment and emission quantum efficiencies, but these are beyond the scope of this study.

## Conclusions

In summary, we used a combination of theoretical and experimental techniques to investigate the structure-emission property relationship of five bilayer (*n* = 2) 2D-PVK crystals adopting either RP- or DJ-phases. Compared with the RP-phase crystals, we found that DJ-phase crystals generally have a higher degree of octahedral lattice distortion that scales with decreasing interlayer spacing. In DJ-phase crystals with short interlayer spacing (such as 4AMP), we found that the top and bottom Pb–I octahedral layers can have largely different and nonuniform bond lengths and angles, giving rise to both intra- and interoctahedral distortions.

Such local intra- and interoctahedral distortions in the DJ-phase crystals can lead to significantly lower formation energies for iodine vacancy point defects in the octahedral lattice that act as nonradiative recombination centers for excitons/carriers, thereby reducing emission intensity. Furthermore, in the case of 4AMP crystal, the large degree of intra- and interoctahedral distortions is sufficient to cause local inversion asymmetry and significant Rashba band splitting, leading to second harmonic generation and circular polarization-dependent PL. Although the circular polarization-dependent PL in the 4AMP crystal is desirable for optoelectronic/spintronic devices, the high iodine vacancy defect density in this crystal also leads to fast trap-assisted nonradiative recombination and thus weak emission intensity at room temperature. Developing strategies to overcome iodine vacancy point defect formation while simultaneously enabling large structural noncentrosymmetry is thus urgently needed to promote the use of 2D-PVKs for optoelectronic/spintronic applications.

## Supplementary Material



## Notes

Details of synthetic procedure, experiments methods, and additional structural and optical characterization data (PDF)
