# Supplementary material for: Structure–Emission Property Relationship of Bilayer 2D Hybrid Perovskites
Source: J Am Chem Soc. 2025 Apr 30;147(23):19902–10. doi: 10.1021/jacs.5c04417 (PMC12164342; doi:10.1021/jacs.5c04417)
Supplement: Supplementary file 1 [file ja5c04417_si_001.pdf]

Supporting Information

Structure-emission property relationship of bilayer 2D hybrid perovskites

Yumeng Song,[1,#] Yifan Zhou,[2,#] Congcong Chen,[3] Kezhou Fan,[4] Zhen Wang,[1] Yu Guo,[1] Ziming Chen,[1] Lingling Mao,[3] Jun Yin,[2,*] Philip C. Y. Chow[1,*]

1. Department of Mechanical Engineering, The University of Hong Kong, Pokfulam, Hong Kong, China
2. Department of Applied Physics, The Hong Kong Polytechnic University, Kowloon, Hong Kong, China
3. Department of Chemistry, Southern University of Science and Technology, Shenzhen, Guangdong, 518055, China
4. Department of Physics, The Hong Kong University of Science and Technology, Clearwater Bay, Hong Kong, China

*Corresponding authors: jun.yin@polyu.edu.hk; pcyc@hku.hk

Experimental Methods

Chemicals and synthesis

Hexylamine (HA, 99%), 4-(aminomethyl) piperidinium (4AMP, 98%) 1,9-Diaminononane (NDA, 98%) and 1,7-Heptanediamine (DAH, 98%) were purchased from Sigma-Aldrich and Tokyo Chemical Industry (TCI). Lead iodine (PbI_2, high purity of 99.99%), CH_3NH_3I (MAI, high purity of 99.99%) and phenylethyl ammonium iodide (PEAI, high purity of 99.99%) were purchased from Great Cell Solar Materials. Hydroiodic acid (57 wt % in H_2O, distilled, stabilized, 99.95%) and hypo phosphorous acid solution (50 wt % in H_2O) were purchased from Thermo Scientific. All chemicals were of analytical grade and directly used without further purification.

- ***(PEA)$_2$(MA)Pb$_2$I$_7$***. PbI_2 (184.4 mg, 0.4 mmol), phenylethylammonium iodide (42.3 mg, 0.17 mmol), and CH_3NH_3I (106.5 mg, 0.67 mmol) were dissolved in the mixture of 1000 µL of 57% w/w aqueous HI solution and 100 µL of 50% aqueous H_3PO_2 by heating to 120 °C under constant magnetic stirring for about 10 min, which formed a bright yellow solution. Afterward, the stirring was discontinued, then the resultant solution was slowly cooled to room temperature (-5°C/20min), and the cherry red rectangular-shaped plates started to crystallize. Stand the solution overnight, then the crystals were isolated by suction filtration and thoroughly dried under reduced pressure.
- ***(HA)$_2$(MA)Pb$_2$I$_7$***. PbI_2 (230.5 mg, 0.5 mmol) and CH_3NH_3I (40 mg, 0.25 mmol) were dissolved in 800 µL of 57% w/w aqueous HI solution by heating to 120 °C under constant magnetic stirring for about 5 min, which formed a bright yellow solution. In a separate beaker, $CH_3(CH_2)_5NH_2$ (29 µL, 0.22 mmol) was neutralized with 100 µL of 50% aqueous H_3PO_2 resulting in a clear yellow solution. The neutralized HA solution

was added to the above HI solution under stirring until a clear yellow solution was obtained. Afterward, the stirring was discontinued, then the resultant solution was slowly cooled to room temperature (-5°C/20min), and the cherry red rectangular-shaped plates started to crystallize. Stand the solution overnight, then the crystals were isolated by suction filtration and thoroughly dried under reduced pressure.

- ***(4AMP)(MA)Pb$_2$I$_7$***. PbI_2 (230.5 mg, 0.5 mmol) and CH_3NH_3I (80 mg, 0.5 mmol) were dissolved in 1300 μL of 57% w/w aqueous HI solution by heating to 120 °C under constant magnetic stirring for about 5 min, which formed a bright yellow solution. In a separate beaker, 4-(aminomethyl) piperidinium (7 μL, 0.055 mmol) was neutralized with 333 μL of 50% aqueous H_3PO_2 resulting in a clear yellow solution. The neutralized 4AMP solution was added to the above HI solution under stirring until a clear yellow solution was obtained. Afterward, the stirring was discontinued, then the resultant solution was slowly cooled to room temperature (-5°C/20min), and the cherry red rectangular-shaped plates started to crystallize. Stand the solution overnight, then the crystals were isolated by suction filtration and thoroughly dried under reduced pressure.
- ***(NDA)(MA)Pb$_2$I$_7$***. PbI_2 (230.5 mg, 0.5 mmol) and CH_3NH_3I (80 mg, 0.5 mmol) were dissolved in 1000 μL of 57% w/w aqueous HI solution by heating to 120 °C under constant magnetic stirring for about 5 min, which formed a bright yellow solution. In a separate beaker, $NH_2(CH_2)_9NH_2$ (23.7mg, 0.15 mmol) was neutralized with 200 μL of 50% aqueous H_3PO_2 resulting in a clear yellow solution. The neutralized NDA solution was added to the above HI solution under stirring until a clear yellow solution was obtained. Afterward, the stirring was discontinued, then the resultant solution was slowly cooled to room temperature (-5°C/20min), and the cherry red rectangular-shaped plates started to crystallize. Stand the solution overnight, then the crystals were isolated by suction filtration and thoroughly dried under reduced pressure.
- ***(DAH)(MA)Pb$_2$I$_7$***. PbI_2 (230.5 mg, 0.5 mmol) and CH_3NH_3I (80 mg, 0.5 mmol) were dissolved in 1000 μL of 57% w/w aqueous HI solution by heating to 120 °C under constant magnetic stirring for about 5 min, which formed a bright yellow solution. In a separate beaker, $NH_2(CH_2)_7NH_2$ (33.2mg, 0.25 mmol) was neutralized with 200 μL of 50% aqueous H_3PO_2 resulting in a clear yellow solution. The neutralized NDA solution was added to the above HI solution under stirring until a clear yellow solution was obtained. Afterward, the stirring was discontinued, then the resultant solution was slowly cooled to room temperature (-5°C/20min), and the cherry red rectangular-shaped plates started to crystallize. Stand the solution overnight, then the crystals were isolated by suction filtration and thoroughly dried under reduced pressure.

Sample exfoliation

We performed mechanical exfoliation on plate-like single crystals and positioned them on a transparent one-sided scotch tape. Another clean section of tape was carefully folded over the crystals, ensuring that a portion of the crystal was detached for subsequent exfoliation, thus revealing fresh cleaved layers. The remaining section of the crystal remained affixed to the tape. This iterative process was repeated several times to achieve the desired optically thin 2D perovskite sheets. Finally, we transferred the exfoliated samples onto a coverslip by gently pressing the tape onto its surface. The exfoliated samples were encapsulated by epoxy glue and coverslip inside nitrogen glovebox to prevent sample degradation during optical experiments. The surface morphologies and thickness of exfoliated samples were examined on a commercial multifunction AFM instrument (Bruker Dimension Icon SPM) in tapping mode using NCHV-A tips from Bruker AFM Probes.

Powder and single-crystal X-ray diffraction

Powder X-ray diffraction (PXRD) data were performed using a Rigaku SmartLab SE X-ray diffractometer (Cu Kα radiation; λ = 1.54056 Å) operating at 45 kV and 200 mA in the range of 5°−50° to confirm the purity of each sample at room temperature. For single-crystal XRD, a clear plate-shaped crystal were mounted on the goniometer inside a Bruker D8 Venture diffractometer with Mo-Kα (λ = 0.71073 Å) radiation at 100 K, respectively. All data were integrated with SAINT and a multi-scan absorption correction using SADABS was applied. The structure was solved by dual methods using SHELXT and refined by full-matrix least-squares methods against F2 by XL using Olex2. All non-hydrogen atoms were refined with anisotropic displacement parameters. All C-bound hydrogen atoms were refined isotropic on calculated positions using a riding model with their Uiso values constrained to 1.5 times the Ueq of their pivot atoms for terminal sp3 carbon atoms and 1.2 times for all other carbon atoms. Disordered moieties were refined using bond lengths restraints and displacement parameter restraints. Crystallographic data for the structures reported here have been deposited onto Cambridge Crystallographic Data Centre.

Absorption and Raman spectroscopy

Steady-state absorption spectra were recorded by a UV-vis absorption spectrometer (Cary60, Agilent Technologies, USA). Low-frequency Raman experiments were performed at a 633 nm beam from a He−Ne laser. 1800 lines/mm and 2400 lines/mm gratings were used in the Raman measurements, where the spectral resolution was 0.19 cm^{-1} per CCD pixel under 633 nm excitation with 2400 lines/mm. The laser plasma lines were removed by Bragg-volume-grating-based bandpass filters from Opti Grate Corp. Acquisitions employed a 10×optical objective and used minimal laser intensity to avoid laser-induced damage. To achieve a suitable signal quality, each wave-number Raman signal was integrated over at least 10 s.

Photoluminescence

A home-built PL microscopy setup based on an Olympus BX53 microscope was used to study the emission properties and dynamics of the exfoliated 2D-PVK samples. Photoexcitations were provided by a picosecond light pulse at 520 nm generated by a SC-PRO supercontinuum source (Wuhan Yangtze Soton Laser Co., Ltd; 1 MHz). The excitation power was measured using a calibrated Newport power meter, and the fluence were calculated based on the beam area fitted with the Gaussian beam model. PL spectra were acquired with a Princeton Instruments (HRS-300) monochromator integrated with a PIXIS CCD camera. Time-resolved PL (TRPL) decays were acquired with a PicoQuant MPD single photon avalanche diode and a PicoHarp-300 timer. TRPL kinetics were probed at the PL peaks for each sample, and the wavelengths of probed PL are selected by the a forementioned HRS-300 spectrograph. A neutral density filter (Thorlabs) was used to control excitation fluence. The circular polarization of the laser was modulated by a linear polarizer and a quarter-waveplate ($\lambda/4$, Thorlabs SAQWP05M-700) then passed through the non-polarizing dichroic mirror, and the circular polarization emission of the sample was identified by a quarter-wave plate ($\lambda/4$, Thorlabs SAQWP05M-700) coupled with a polarizer.

Second harmonic generation

Second harmonic generation (SHG) measurements were conducted by a home-built microscope setup. A Titanium Sapphire regenerative amplifier (Coherent Legend Elite, 1000 Hz, 100 fs) is seeded by a titanium sapphire oscillator (Coherent Mira 900, fundamental wavelength ~800 nm). The output of the amplifier is directed into an optical parametric amplifier (OPA, Coherent Opera Solo) which provides the pump beam with tunable photon energy. The output laser wavelength is tunable and is focused by an objective (Olympus, ×20,

NA = 0.4) onto the surface of the sample. The generated SH is collected by the same objective and is separated from the fundamental wave by a dichroic mirror, which transmits light with wavelength longer than 950 nm while reflecting short-wavelength light. The SHG is directed to a spectrometer (Acton Spectrapro 275) equipped with a 150 ln/mm grating and coupled to a CCD. A 900 nm short-pass filter is positioned before the fiber to spectrometer to minimize the fundamental harmonic.

Density functional theory (DFT) calculations

Structural optimizations. The crystal structures obtained from single-crystal X-ray diffraction experiments were optimized using projector-augmented wave (PAW) method with generalized gradient approximation (GGA), Perdew-Burke-Ernzerhof (PBE) exchange correlation functional, and enhanced with D3(BJ) dispersion correlation as implemented in Vienna Ab initio Simulation Package. This optimization aims to obtain a more stable and accurate configuration, addressing potential discrepancies that may arise from experimental variations. A plane-wave cut-off energy of 450 eV and a Monkhorst-Pack grid of *k*-points with the smallest allowed spacing of 0.03 Å was employed. The Gaussian smearing was set to be 0.05 eV, and the self-consist field (SCF) energy convergence criterion was set as 1×10^{-6} eV with the structural energy convergence criterion set to 0.02 eV/Å. The energy cutoff for plane wave expansion maintained at 450 eV, while other settings remained the consistent with *k*-points at the Γ-point. The simulated PXRD patterns of the DFT-optimized structures were obtained from the powder diffraction pattern utility using the Mercury software. Geometrical parameters is derived by averaging the values across all eight Pb–I octahedra in the supercell representation of both top (L1) and bottom (L2) layers for each bilayer crystal (Fig. S4).

Electronic band calculations. The GGA/PBE functional with spin-orbital coupling (SOC) was employed for the electronic band structure calculation. The Brillion zone, from which the high-symmetry *k*-points are derived, was generated from the convention cell for all the crystals. The energy cutoff for the plane wave expansion was set to be 450 eV and SCF energy convergence criterion was set to 1×10^{-6} eV.

Defect formation energy calculations. The 2×2×1 supercells were employed for all crystals for the defect formation energy calculations. The formation energies of three types of point defects were considered, including in-plane iodine vacancies ($V_{I\text{-}in}$), out-of-plane iodine vacancies ($V_{I\text{-}out}$), and organic spacer vacancies (V_{Spacer}) for all crystals. The defects formation energies were calculated by using the following formula:

$$\Delta H_{defect} = E_{defect} - E_{pristine} + \sum_i n_i\,(\mu_i + E_i)$$

where $\Delta H_{\mathrm{defect}}$ is the defect formation energy, E_{defect} and E_{pristine} are the total energies of the defected and pristine structure, respectively. n_{i} is the number of species *i* removed from the crystal structure ($n_{\mathrm{i}} > 0$). μ_{i} and E_{i} are the chemical potential and elemental solid energy of specie *i*, respectively. To determine the feasible chemical potential regions for thermal equilibrium growth of these bilayer 2D-PVK crystals, the chemical potential of Pb, I, organic spacer and MA should satisfy the following relationship, ensuring the formation of these crystals and preventing the formation of secondary compounds such as PbI_2, MAI, and corresponding single layer 2D-PVK with the same organic spacer:

$$n\mu_{Spacer} + 2\mu_{Pb} + 7\mu_I + \mu_{MA} = \Delta H(Spacer_n MAPb_2I_7)$$

$$n\mu_{Spacer} + \mu_{Pb} + 4\mu_I < \Delta H(Spacer_n PbI_4)$$
$$\mu_{Pb} + 2\mu_I < \Delta H(PbI_2)$$
$$\mu_{MA} + \mu_I < \Delta H(MAI)$$

where n is the stoichiometric coefficient for organic spacer (n = 1 for 4AMP, NDA, AEPA, DAH and HDA, and n = 2 for PEA and HA). The acquired feasible chemical potential regions are 3D spaces spanned by chemical potential of Pb, I and spacer. Three different representative points were sampled, representing three different growth conditions: Pb-poor/I-rich, Moderate, and Pb-rich/I-poor within the feasible chemical region of each crystal for the defect formation energy calculations.

Supporting Note: Estimation of exciton/carrier density in PL measurements

The photogenerated exciton/carrier density (N_0) is determined by the pulsed laser excitation fluence, the photon energy of the excitation pulses, and absorption properties of the crystal. By applying the Beer-Lambert Law, the exciton/carrier concentration can be estimated using the following relationship:

$$N_0 = I \cdot (1 - e^{-\alpha \cdot d}) \cdot (1 - R) \cdot \frac{\lambda}{hc} \cdot \frac{1}{d}$$

where I is the excitation fluence; λ is the wavelength of laser beam (*i.e.*, 520 nm); h is the Plank constant; c is the speed of light; R represents the reflectance of the sample to the laser beam, which is estimated to be 0.2 (*J. Am. Chem. Soc.* **2020,** *142* (35), 15091-15097, *J. Am. Chem. Soc.* **2020,** *142* (26), 11486-11496, and *J. Phys. Chem. Lett.* **2019,** *10* (17), 5153-5159). d represents the effective absorption depth of the 2D perovskite. For a flake thickness of approximately ~300-400 nm (Figure S2), and considering that only the inorganic layers contribute to light absorption, the effective absorption depth is estimated to be ~218 nm. This value is derived by calculating the combined thickness of two inorganic layers (~1.2 nm in total) and one organic layer (~1 nm); The absorption coefficient (α) is estimated to be 1×10^{-4} cm^{-1}, calculated using the equation $\alpha = \frac{A}{d}$, where A = 0.22 is the crystal absorbance at 520 nm. Accordingly, under an excitation fluence of 0.3 μJ cm^{-2}, the generated exciton/carrier density is estimated to be 5.6×10^{15} cm^{-3}, which lies within the first-order recombination regime. At a higher excitation fluence of 3 μJ cm^{-2}, the exciton/carrier density increases to 5.6×10^{16} cm^{-3}, where second-order recombination begins to dominate the overall recombination.[1,2]

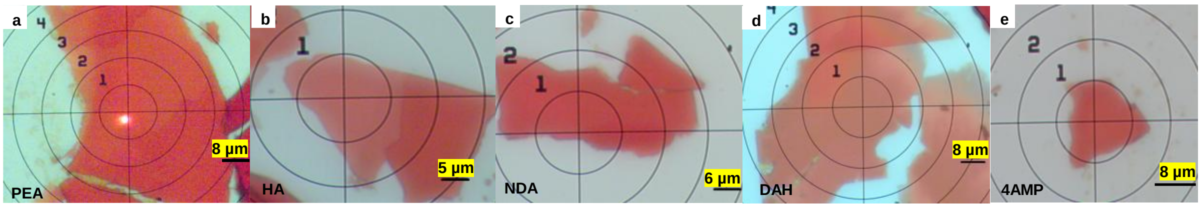

Figure S1. (a–e) Transmitted optical images of the bilayer 2D-PVK crystals. The bright dot in (a) demonstrates the laser spot size of around 3.5 μm. To minimize the influence of surface states at the edges, we ensured excitation at the center of each crystal.

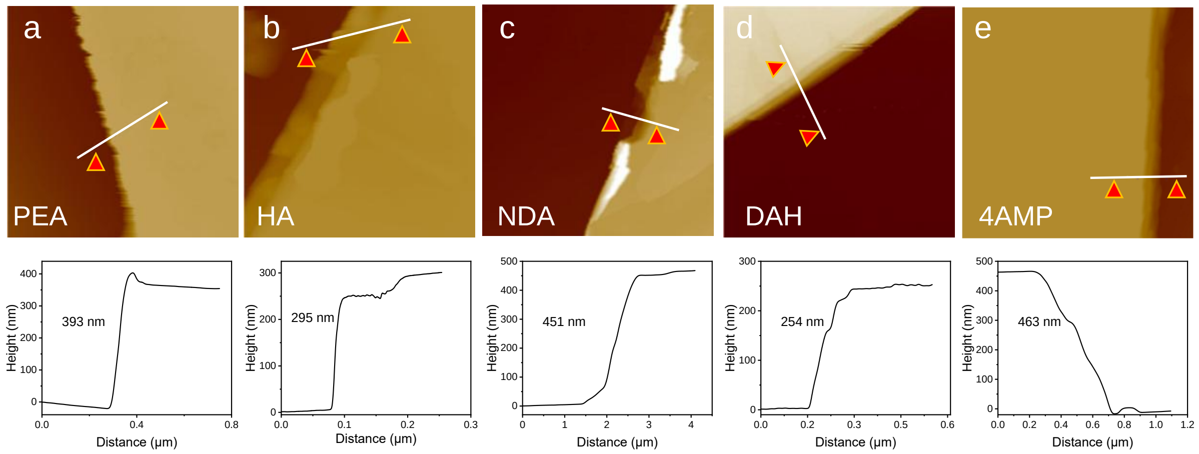

Figure S2. AFM images and height profiles extracted along the white dashed line of exfoliated single crystals of (a) PEA, (b) HA, (c) NDA, (d) DAH, and (e) 4AMP.

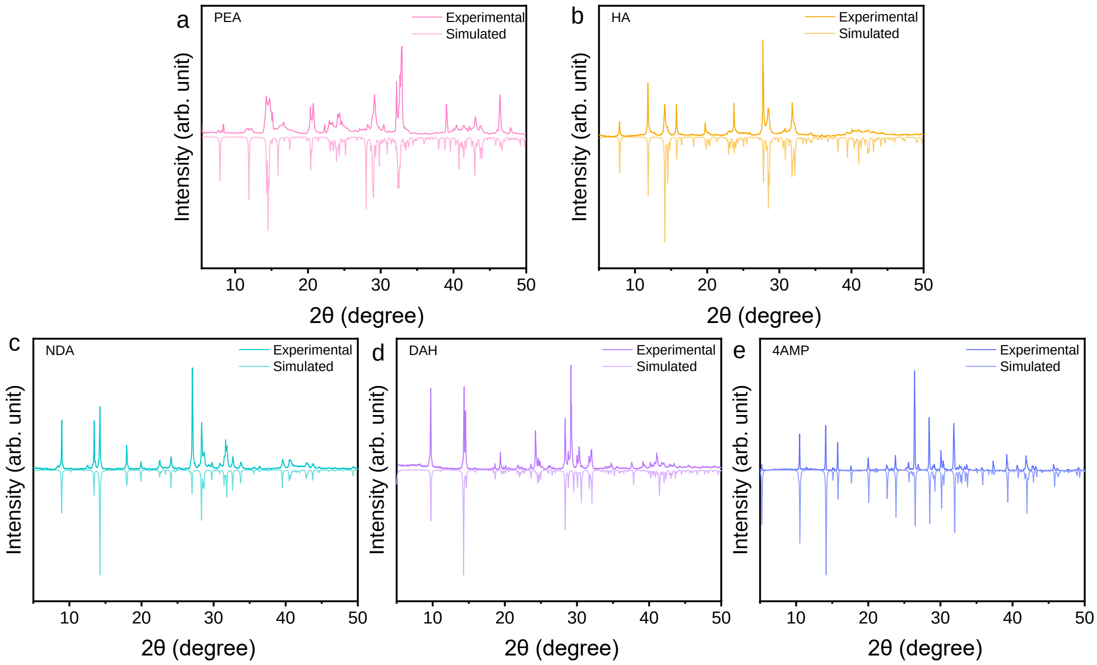

Figure S3. Comparison between experimental PXRD patterns and the simulated PXRD patterns of all crystal structures optimized by DFT. (a) PEA, (b) HA, (c) NDA, (d) DAH, and (e) 4AMP.

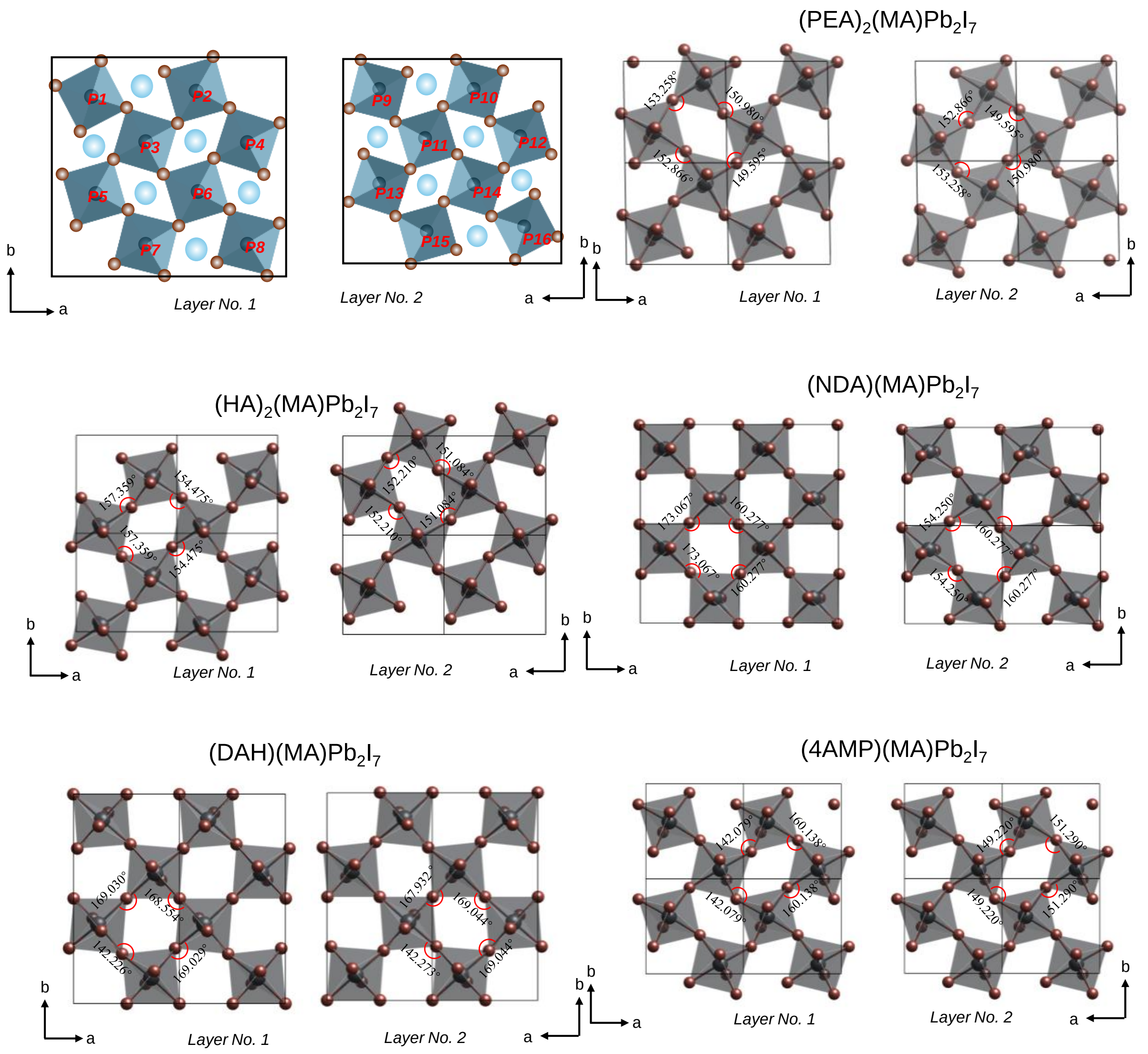

Figure S4. Geometrical parameters are derived by averaging the values across all eight Pb-I octahedra in the supercell representation of both top (L1: P1 to P8) and bottom (L2: P9 to P16) layers for each bilayer 2D-PVK crystal in the supercell representation.

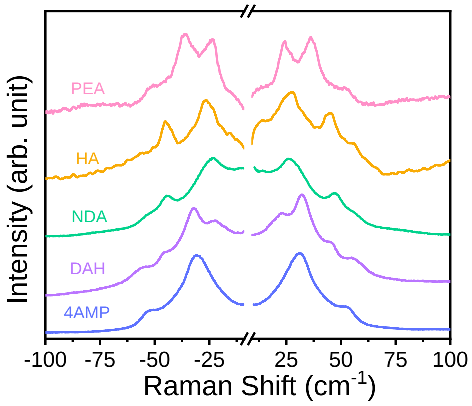

Figure S5. Low-frequency Raman spectra of various bilayer 2D-PVK crystals studied herein.

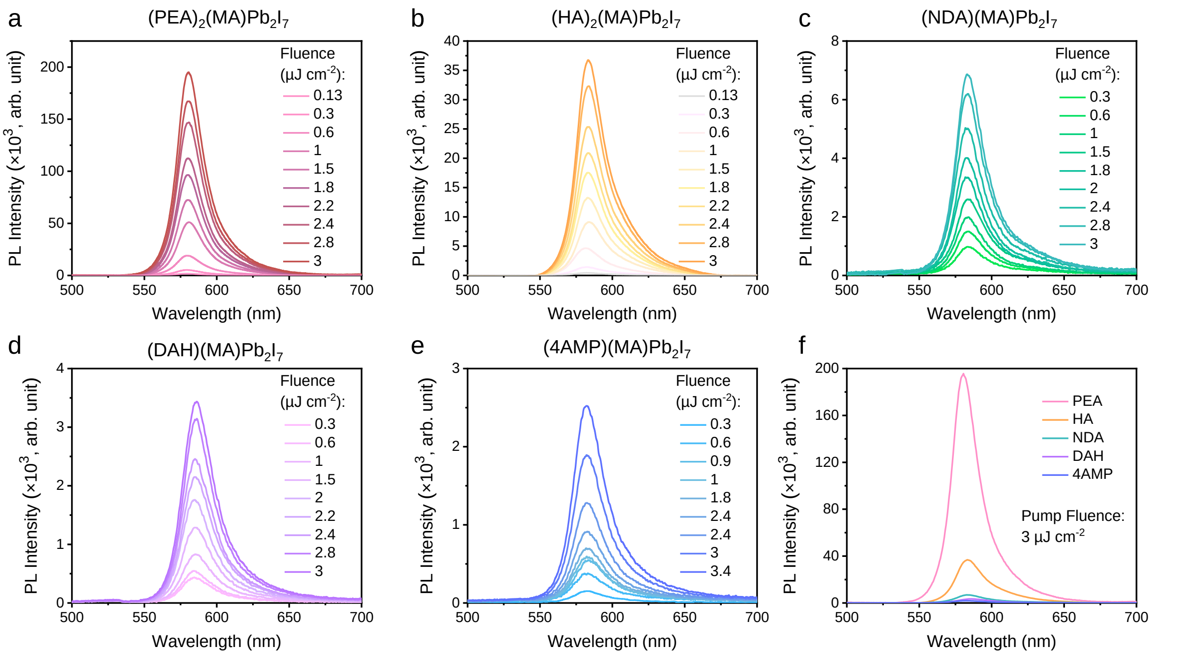

Figure S6. Fluence-dependent PL intensity of bilayer 2D-PVK crystals of (a) $(PEA)_2(MA)Pb_2I_7$, (b) $(HA)_2(MA)Pb_2I_7$, (c) $(NDA)(MA)Pb_2I_7$, (d) $(DAH)(MA)Pb_2I_7$, (e) $(4AMP)(MA)Pb_2I_7$. (f) Their PL spectrum under the pump fluence of 3 μJ cm^{-2}. All the samples were excited by a 520-nm laser.

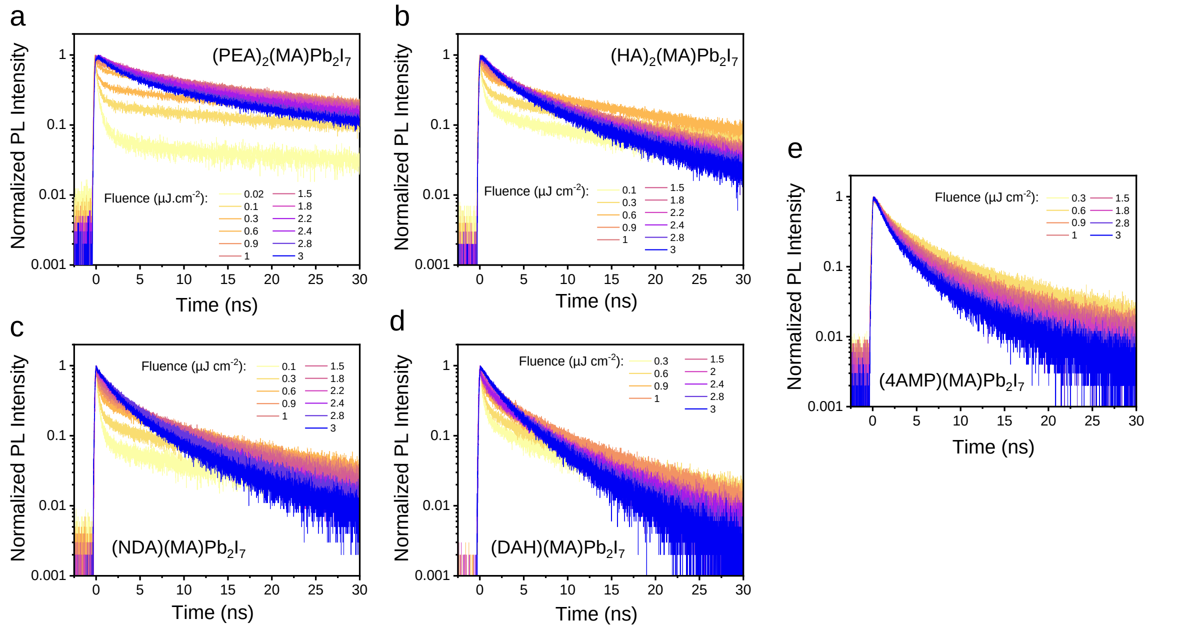

Figure S7. Fluence-dependent TRPL of bilayer 2D-PVK crystals: (a) $(PEA)_2(MA)Pb_2I_7$, (b) $(HA)_2(MA)Pb_2I_7$, (c) $(NDA)(MA)Pb_2I_7$, (d) $(DAH)(MA)Pb_2I_7$, (e) $(4AMP)(MA)Pb_2I_7$.

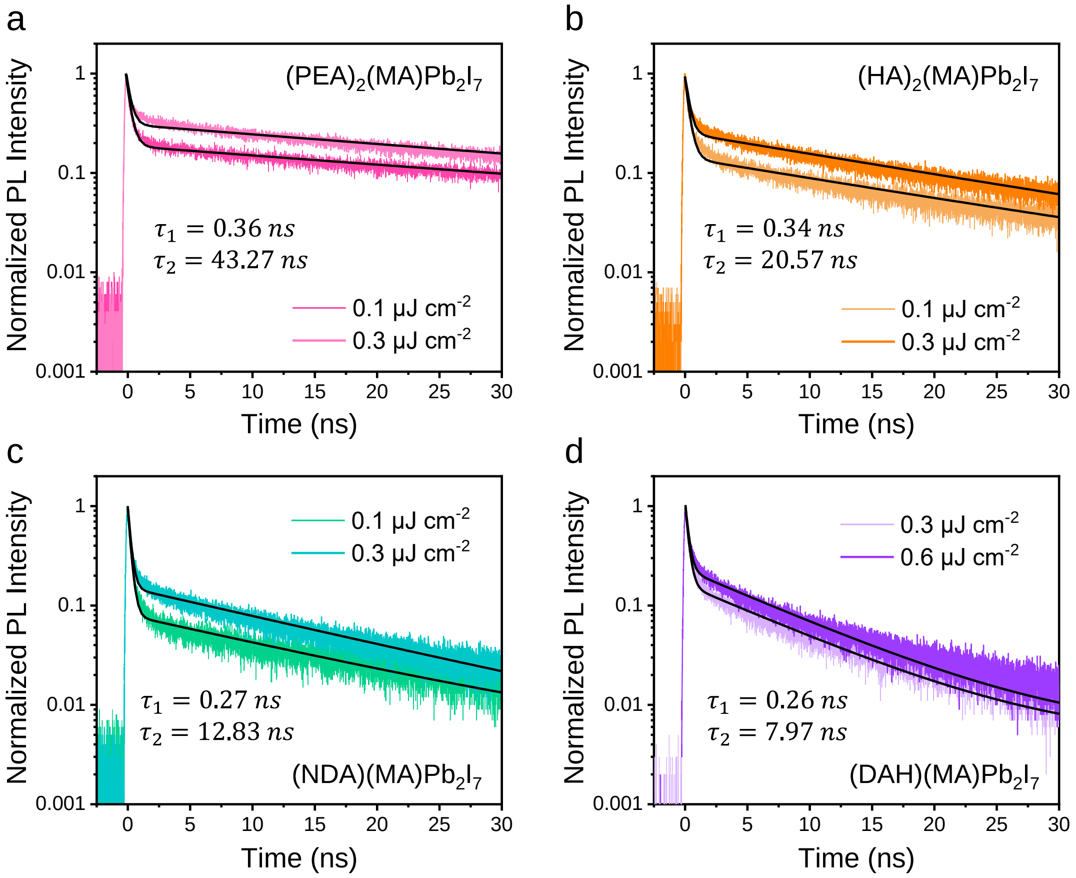

Figure S8. Global fit (black solid curves) of low-pump-fluence TRPL data of (a) $(PEA)_2(MA)Pb_2I_7$, (b) $(HA)_2(MA)Pb_2I_7$, (c) $(NDA)(MA)Pb_2I_7$, (d) $(DAH)(MA)Pb_2I_7$, using bi-exponential decay. Under low pump fluences, all systems are still dominated by a first-order recombination process, as evidenced by the similar decay time of the slow component in each sample. Additionally, we can assume that the trap filling rate remains unchanged since all trap states are far from being completely filled under low-fluence excitation. In such a scenario, the time constants of τ_1 and τ_2 respectively remain identical in various (low) fluences, with only their ratio changing. Therefore, a global fit of low-pump-fluence TRPL data with fixed τ_1 and τ_2 can provide a more precise extraction of them.

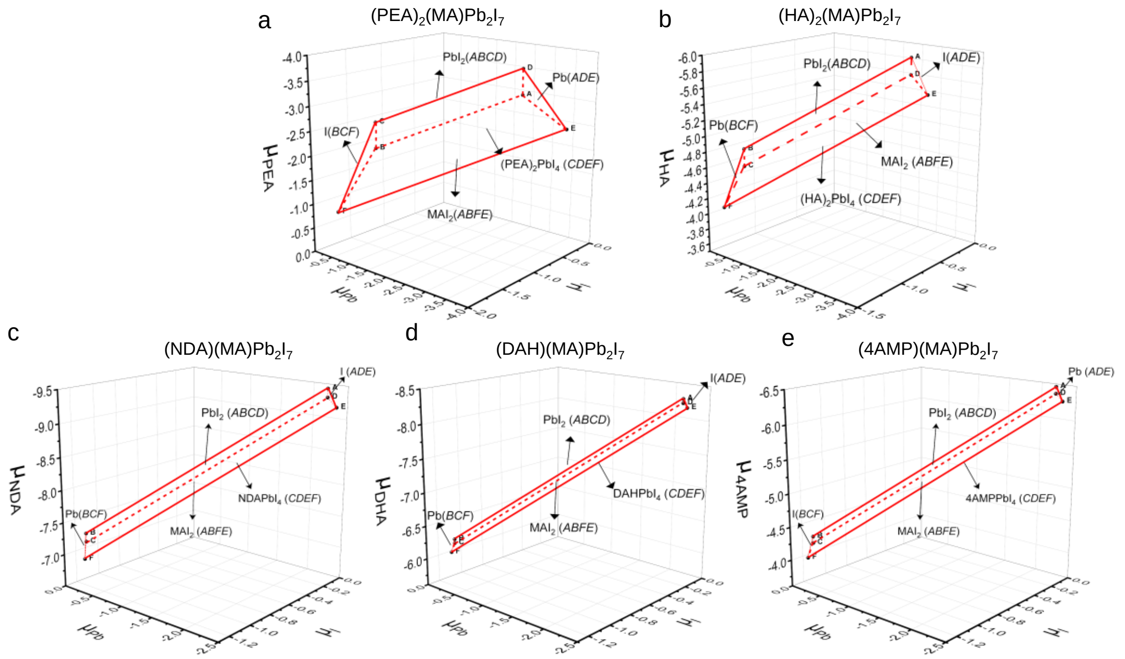

Figure S9. The allowed chemical potential region for the thermal equilibrium growth of various bilayer 2D-PVKs marked by the 3D polyhedral region, as calculated at GGA/PBE+vdW level. (a) $(PEA)_2(MA)Pb_2I_7$, (b) $(HA)_2(MA)Pb_2I_7$, (c) $(NDA)(MA)Pb_2I_7$, (d) $(DAH)(MA)Pb_2I_7$.

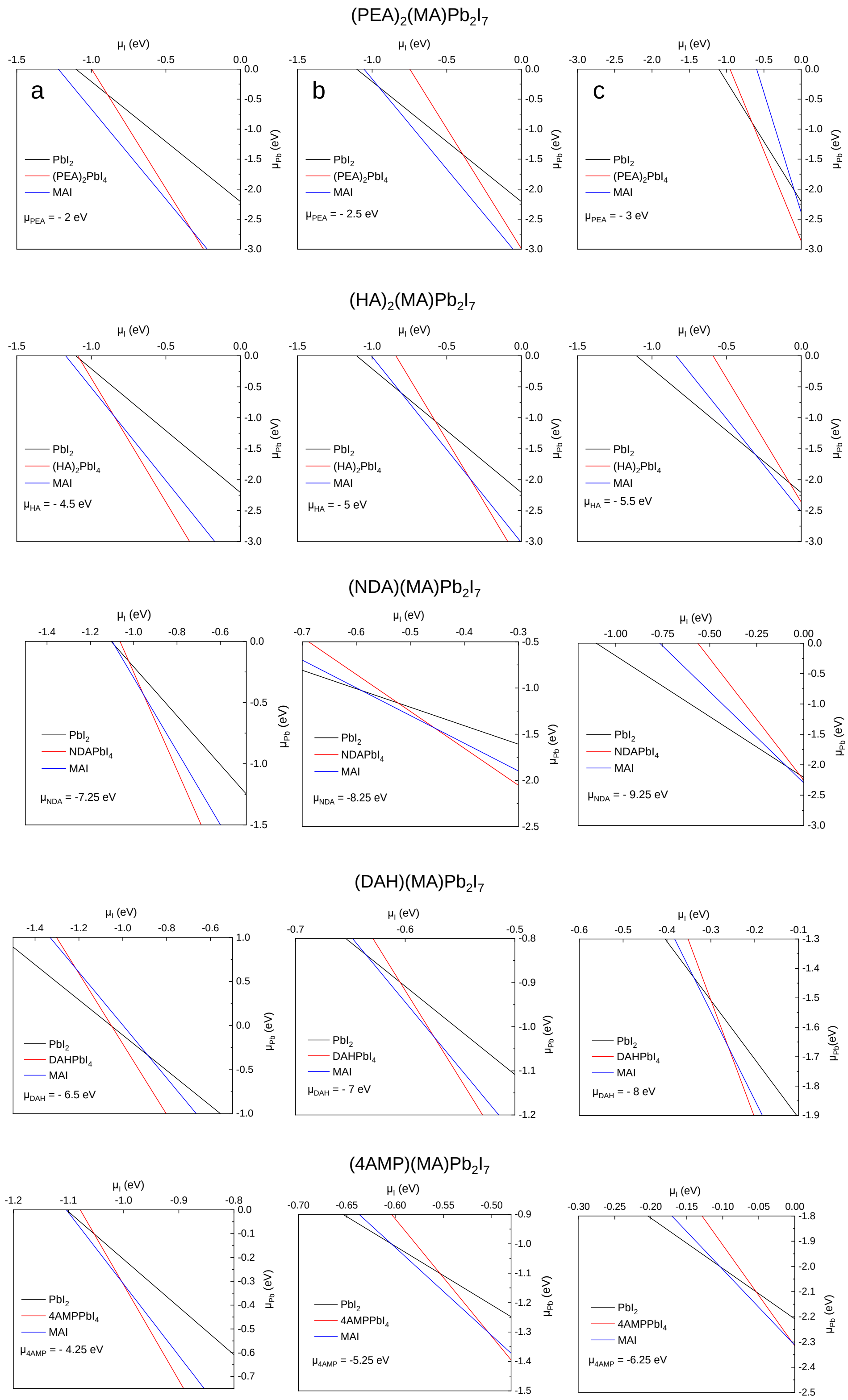

Figure S10. Three different conditions chosen for defects formation energy calculation for various bilayer 2D-PVKs. Three different conditions sliced at (a) Pb-rich, (b) moderate, and (c) I-rich, as labeled in the plots.

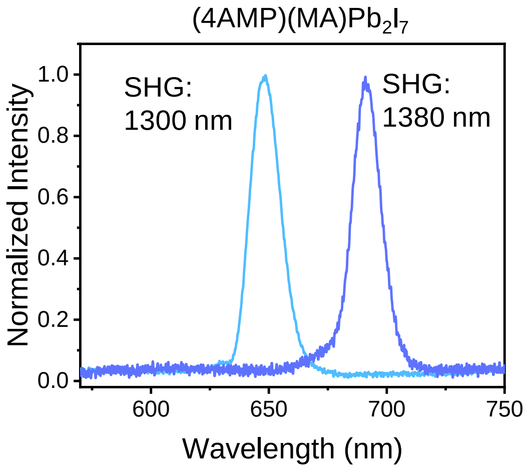

Figure S11. Second harmonic generation (SHG) for the 4AMP crystal, excited by 1300 nm or 1380 nm laser pulses.

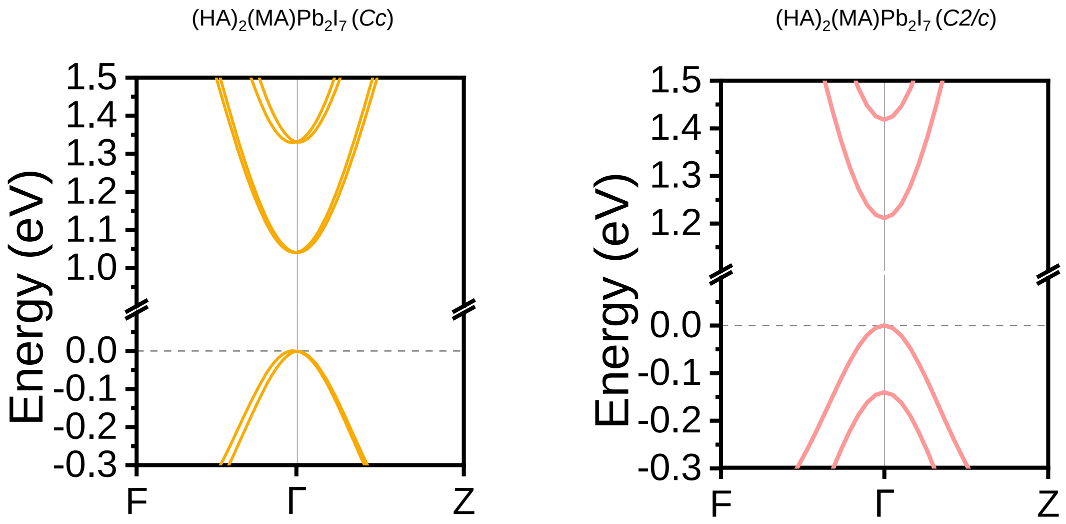

Compound	Space Group	Interlayer Distance (Å)	Layer No.	Avg. Distortion Index ($\times 10^{-2}$)	Avg. σ^2 $(°)^2$	Avg. Quadratic Elongation	Max. $\Delta\beta$(°)	Max. $\Delta\beta_{in}$(°)	Max. $\Delta\gamma$ (°)	Max. $\Delta\gamma_{in}$(°)
$(HA)_2(MA)Pb_2I_7$	*Cc*	9.223	1	1.401	14.336	1.004	0.689	0.852	0.441	0.851
			2	0.359	6.827	1.002	1.126	0.066	0.841	0.308
$(HA)_2(MA)Pb_2I_7$	*C2/c*	9.634	1	0.921	11.033	1.003	4.505	4.463	2.902	3.535
			2	0.923	11.034	1.003	4.507	4.465	2.902	3.531

Figure S12. Electronic band structure and structural analysis for HA crystal using reported CIF for either *Cc* or *C2/c* global space groups.

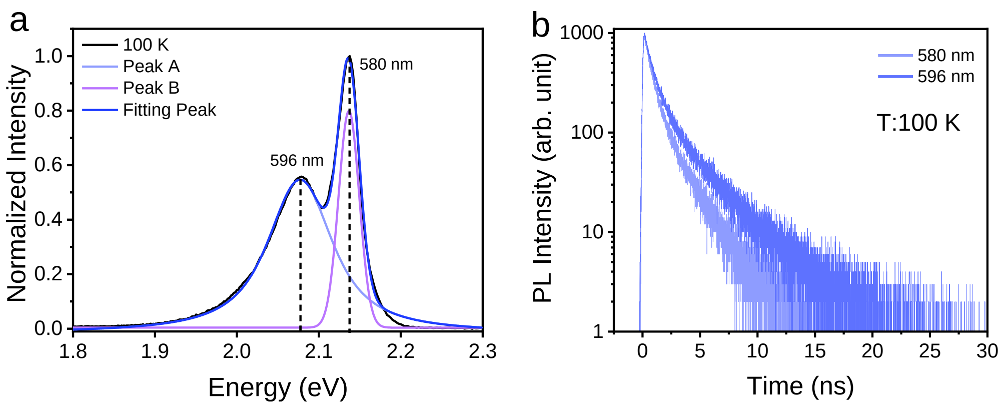

Figure S13. (a) Gaussian peak fitting of PL spectrum of 4AMP crystal at 100 K under 520 nm excitation. (b) TRPL decay kinetics monitored near the peak of the two features, showing different decay rates. No PL spectral splitting is observed at low temperatures for the other crystals involved in this study.

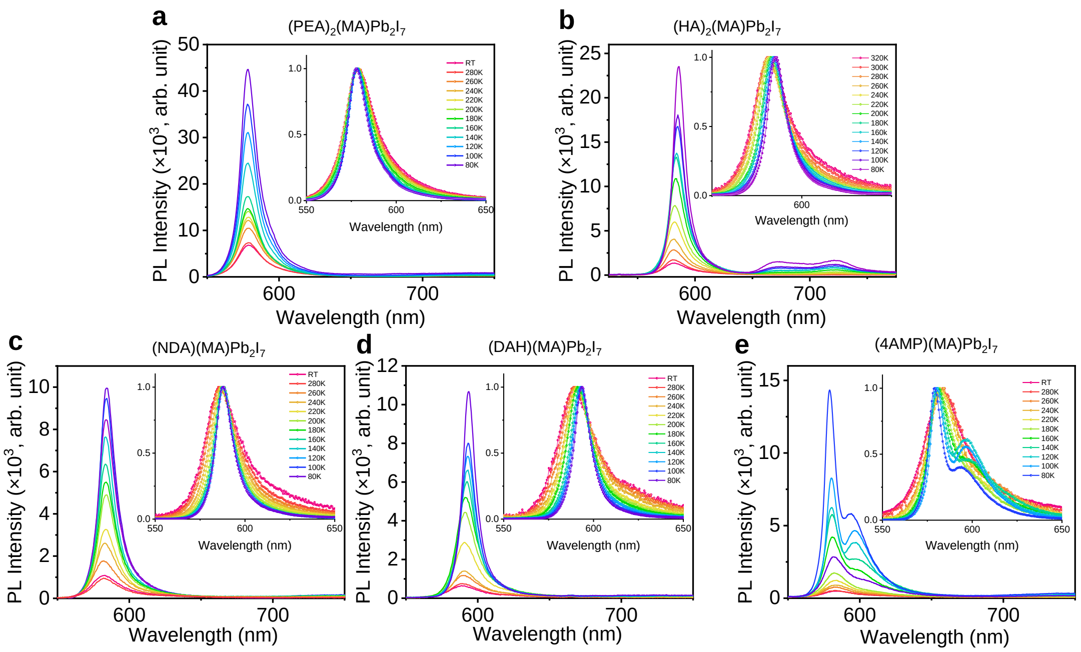

Figure S14. Low temperature photoluminescence spectra for various bilayer 2D-PVK crystals under 520 nm excitation at ~1 μJ cm^{-2}. (a) PEA, (b) HA, (c) NDA, (d) DAH, and (e) 4AMP. Note that the PL intensity is presented in arbitrary units due to variations in camera exposure time during measurements of different samples.

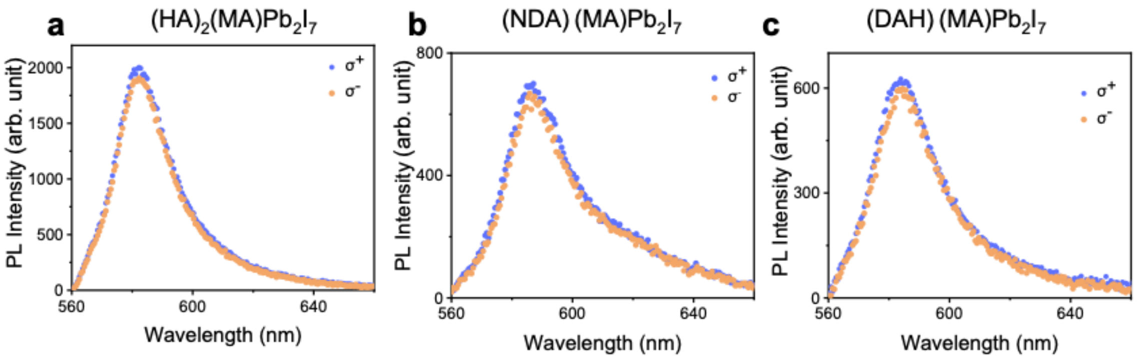

Figure S15. Left (σ-) and right-handed (σ+) circularly-polarized photoluminescence of HA, NDA and DAH crystals under right-handed (σ+) circularly-polarized excitation at 520 nm. Note that the PL intensity is presented in arbitrary units due to variations in camera exposure time during measurements of different samples.

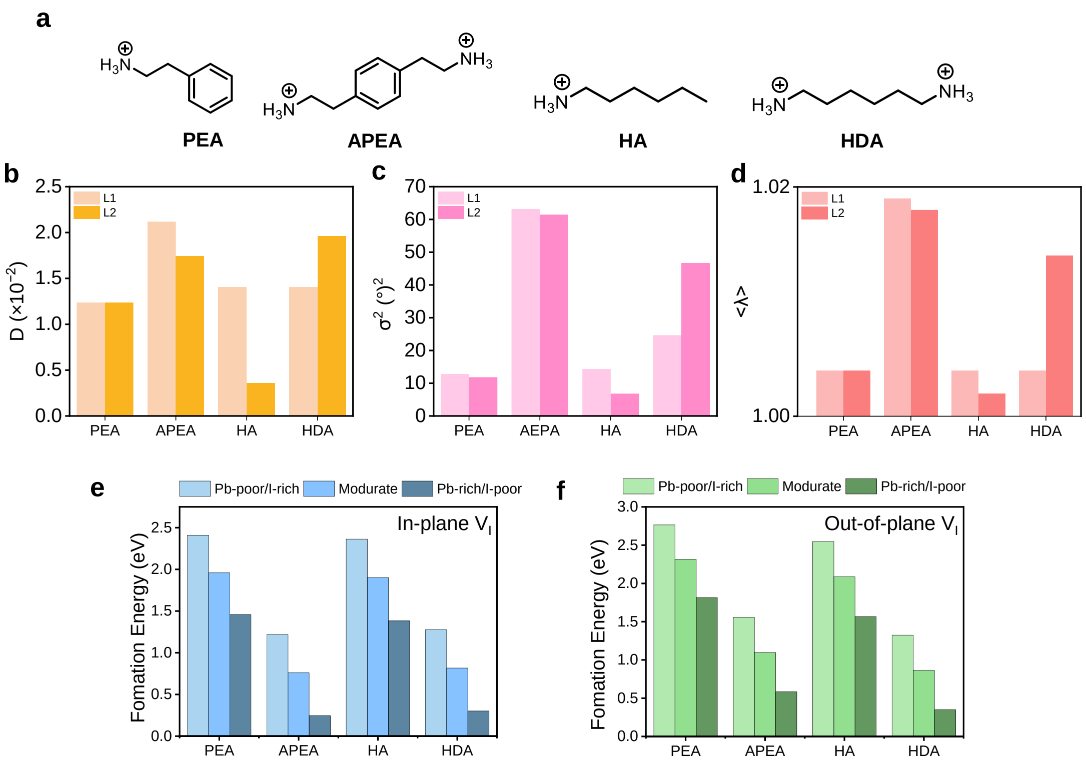

Figure S16. (a) Molecular structure of PEA, APEA, HA and HDA. Comparison of the (b) distortion index (*D*), (c) bond angle variance (σ^2), and (d) quadratic elongation (<λ>) of top/bottom octahedral layers for these four bilayer 2D-PVKs. (e) in-plane V_I and (f) out-of-plane V_I in bilayer 2D-PVK single crystals based on PEA, APEA, HA, and HDA, as calculated at GGA/PBE+vdW level.

Identification code	2422656
Empirical formula	$C_8H_{26}I_7N_3Pb_2$
Formula weight	1467.00
Temperature/K	100.00
Crystal system	orthorhombic
Space group	Ibam
a/Å	8.9590(5)
b/Å	17.6548(9)
c/Å	36.243(2)
α/°	90
β/°	90
γ/°	90
Volume/Å^3	5732.5(5)
Z	2
ρ_{calc} g/cm^3	3.400
μ/mm^{-1}	19.270
F (000)	5040.0
Radiation	MoKα (λ = 0.71073)
2Θ range for data collection/°	4.496 to 50.698
Index ranges	-10 ≤ h ≤ 9, -21 ≤ k ≤ 20, -36 ≤ l ≤ 43
Reflections collected	11057
Independent reflections	2667 [R_{int} = 0.0491, R_{sigma} = 0.0434]
Data/restraints/parameters	2667/235/138
Goodness-of-fit on F^2	1.100
Final R indexes [I>=2σ (I)]	R_1 = 0.0317, wR_2 = 0.0601
Final R indexes [all data]	R_1 = 0.0468, wR_2 = 0.0665
Largest diff. peak/hole / e Å^{-3}	1.26/-1.19

Table S1. CIF for the bilayer DAH crystal extracted from original single-crystal X-ray diffraction data. The CIF has been uploaded onto Cambridge Crystallographic Data Centre.

Compound	Layer No.	β (°) ($\beta_1, \beta'_1, \beta_2, \beta'_2$)	β_{in} (°) ($\beta_{1in}, \beta'_{1in}, \beta_{2in}, \beta'_{2in}$)	γ (°) ($\gamma_1, \gamma'_1, \gamma_2, \gamma'_2$)	γ_{in} (°) ($\gamma_{1in}, \gamma'_{1in}, \gamma_{2in}, \gamma'_{2in}$)
(4AMP)(MA)Pb_2I_7	1	142.079, 160.138<br>142.079, 160.138	142.208, 160.002<br>142.208, 160.002	82.309, 97.696<br>93.521, 85.695	92.037, 83.870<br>100.942, 83.148
	2	149.220, 151.290<br>149.220, 151.290	149.336,151.406<br>149.336,151.406	91.061, 88.994<br>90.302, 89.641	92.701, 90.631<br>87.983, 88.683
$(HA)_2$(MA)Pb_2I_7	1	157.359, 156.670<br>157.359, 156.670	157.548,158.400<br>157.548,158.400	89.075, 89.109<br>90.327, 90.768	88.599, 88.862<br>91.694, 90.843
	2	152.210, 151.084<br>152.210, 151.084	152.078, 152.144<br>152.078, 152.144	90.237, 91.041<br>89.186, 89.199	88.631, 88.939<br>91.246, 91.181
(NDA)(MA)Pb_2I_7	1	173.067, 160.277<br>173.067, 160.277	176.122, 177.588<br>176.122, 177.588	89.759, 91.191<br>89.722, 88.499	90.720, 89.253<br>90.159, 89.866
	2	154.250, 154.475<br>154.250, 154.475	161.965, 168.264<br>161.965, 168.264	87.629, 88.795<br>88.707, 94.783	86.781, 93.080<br>91.057, 89.080
$(PEA)_2$(MA)Pb_2I_7	1	152.866, 149.595<br>150.980, 153.258	153.228, 150.310<br>151.002, 153.911	89.104, 91.580<br>91.144, 89.281	91.508, 87.799<br>90.831, 88.485
	2	152.866, 149.595<br>150.980, 153.258	153.228, 150.310<br>151.002, 153.911	89.104, 91.580<br>91.144, 89.281	91.508, 87.799<br>90.831, 88.485
(APEA)(MA)Pb_2I_7	1	154.946, 154.078<br>159.595, 154.347	157.741, 160.654<br>160.183, 162.479	87.549, 88.072<br>90.092, 86.998	87.946, 89.864<br>90.280, 87.644
	2	156.457, 153.470<br>151.789, 151.701	158.766, 159.347<br>153.425, 161.063	91.347, 87.958<br>93.236, 87.408	91.939, 89.790<br>93.803, 88.094
(HDA)(MA)Pb_2I_7	1	148.830, 156.191<br>160.022, 144.468	150.734, 156.192<br>162.634, 146.815	85.607, 95.313<br>85.125, 91.105	86.115, 95.483<br>85.224, 90.655
	2	145.288, 156.953<br>150.294, 165.927	153.978, 158.643<br>150.294, 168.566	91.081, 92.352<br>84.606, 98.110	91.072, 92.907<br>84.584, 97.675
(DAH)(MA)Pb_2I_7	1	169.029, 142.226,<br>168.554, 169.030	170.251, 173.644,<br>177.230, 170.251	87.888, 89.156,<br>91.453, 89.911	91.508, 91.369,<br>92.361, 88.346
	2	142.273, 169.044,<br>169.044, 167.932	173.649, 170.320,<br>170.320, 176.879	87.953, 89.895,<br>91.302, 88.957	91.553, 88.334,<br>92.146, 91.196
(OHA)(MA)Pb_2I_7	1	164.669, 151.004<br>164.669, 151.004	173.044, 158.114<br>173.044, 158.114	87.174, 98.380<br>90.567, 83.736	87.957, 97.379<br>92.212, 82.449
	2	148.543, 155.584<br>148.543, 155.584	158.522, 162.008<br>158.522, 162.008	89.084, 93.280<br>87.526, 89.901	91.443, 91.676<br>88.690, 88.189

Table S2. Calculated structural parameters Pb–I–Pb bond angle (*β*), in-plane Pb–I–Pb projection (β_{in}), I–Pb–I bond angle (γ), in-plane I–Pb–I projection (γ_{in}) in both top and bottom Pb-I octahedral layers from DFT-optimized crystal structures of bilayer 2D perovskites.

Compound	Distortion Metrics	P1	P2	P3	P4	P5	P6	P7	P8	Avg.	Sdv.
$(4AMP)(MA)Pb_2I_7$	Average Bond Length (Å)	3.238	3.238	3.238	3.238	3.238	3.238	3.238	3.238	3.238	0
	Polyhedral volume (Å^3)	44.349	44.349	44.349	44.349	44.349	44.349	44.349	44.349	44.349	0
	Distortion index (×10^{-2})	2.504	2.502	2.504	2.502	2.505	2.502	2.505	2.502	2.503	0.001
	Bond angle variance σ^2 (°^{2})	50.699	50.702	50.699	50.702	50.683	50.687	50.683	50.687	50.693	0.008
	Quadratic elongation	1.015	1.015	1.015	1.015	1.015	1.015	1.015	1.015	1.015	0
	Effective coordination number	5.822	5.822	5.822	5.822	5.822	5.822	5.822	5.822	5.822	0
$(HA)_2(MA)Pb_2I_7$	Average Bond Length (Å)	3.216	3.216	3.216	3.216	3.216	3.216	3.216	3.216	3.216	0
	Polyhedral volume (Å^3)	44.094	44.096	44.094	44.096	44.094	44.096	44.094	44.096	44.095	0.001
	Distortion index (×10^{-2})	1.401	1.401	1.401	1.401	1.401	1.401	1.401	1.401	1.401	0
	Bond angle variance σ^2 (°^{2})	14.335	14.336	14.335	14.336	14.335	14.336	14.335	14.336	14.336	0.001
	Quadratic elongation	1.004	1.004	1.004	1.004	1.004	1.004	1.004	1.004	1.004	0
	Effective coordination number	5.921	5.921	5.921	5.921	5.921	5.921	5.921	5.921	5.921	0
$(NDA)(MA)Pb_2I_7$	Average Bond Length (Å)	3.195	3.195	3.195	3.195	3.195	3.195	3.195	3.195	3.195	0
	Polyhedral volume (Å^3)	43.302	43.301	43.302	43.301	43.303	43.302	43.303	43.302	43.302	0.001
	Distortion index (×10^{-2})	2.425	2.427	2.425	2.427	2.426	2.426	2.426	2.426	2.426	0.001
	Bond angle variance σ^2 (°^{2})	9.111	9.110	9.111	9.110	9.109	9.109	9.109	9.109	9.110	0.001
	Quadratic elongation	1.004	1.004	1.004	1.004	1.004	1.004	1.004	1.004	1.004	0
	Effective coordination number	5.689	5.689	5.689	5.689	5.689	5.689	5.689	5.689	5.689	0
$(PEA)_2(MA)Pb_2I_7$	Average Bond Length (Å)	3.197	3.196	3.197	3.196	3.197	3.196	3.197	3.196	3.197	0
	Polyhedral volume (Å^3)	43.307	43.332	43.307	43.332	43.307	43.332	43.307	43.332	43.320	0.013
	Distortion index (×10^{-2})	1.163	1.305	1.162	1.305	1.163	1.305	1.162	1.305	1.234	0.071
	Bond angle variance σ^2 (°^{2})	13.998	11.532	13.995	11.531	13.998	11.532	13.995	11.531	12.764	1.232
	Quadratic elongation	1.004	1.004	1.004	1.004	1.004	1.004	1.004	1.004	1.004	0
	Effective coordination number	5.953	5.933	5.953	5.933	5.953	5.933	5.953	5.933	5.943	0.010
$(DAH)(MA)Pb_2I_7$	Average Bond Length (Å)	3.210	3.212	3.210	3.212	3.210	3.212	3.210	3.212	3.211	0.001
	Polyhedral volume (Å^3)	43.539	43.590	43.540	43.589	43.539	43.590	43.540	43.589	43.565	0.025
	Distortion index (×10^{-2})	1.914	1.851	1.915	1.853	1.914	1.851	1.915	1.853	1.883	0.031
	Bond angle variance σ^2 (°^{2})	30.265	32.417	30.258	32.411	30.265	32.417	30.263	32.411	31.339	1.076
	Quadratic elongation	1.009	1.010	1.009	1.010	1.009	1.010	1.009	1.010	1.010	0
	Effective coordination number	5.847	5.891	5.847	5.891	5.847	5.891	5.847	5.891	5.869	0.022

Table S3. Structural metrics for each Pb-I octahedra including average bond length, polyhedral volume, distortion index (D), quadratic elongation, bond angle variance, effective coordination number in the top Pb-I octahedral layer (L1) for various bilayer 2D-PVKs.

Compound	Distortion Metrics	P9	P10	P11	P12	P13	P14	P15	P16	Avg.	Sdv.
$(4AMP)(MA)Pb_2I_7$	Average Bond Length (Å)	3.221	3.221	3.221	3.221	3.221	3.221	3.221	3.221	3.221	0
	Polyhedral volume (Å^3)	44.492	44.493	44.492	44.493	44.49	44.493	44.49	44.493	44.492	0.001
	Distortion index (×10^{-2})	0.586	0.586	0.586	0.586	0.585	0.587	0.585	0.587	0.586	0.001
	Bond angle variance σ^2 (°^{2})	2.407	2.410	2.407	2.410	2.407	2.41	2.407	2.4100	2.409	0.001
	Quadratic elongation	1.001	1.001	1.001	1.001	1.001	1.001	1.001	1.001	1.001	0
	Effective coordination number	5.992	5.992	5.992	5.992	5.992	5.992	5.992	5.992	5.992	0
$(HA)_2(MA)Pb_2I_7$	Average Bond Length (Å)	3.229	3.229	3.229	3.229	3.229	3.229	3.229	3.229	3.229	0
	Polyhedral volume (Å^3)	44.748	44.748	44.748	44.748	44.748	44.748	44.748	44.748	44.748	0
	Distortion index (×10^{-2})	0.360	0.359	0.360	0.359	0.360	0.359	0.360	0.359	0.359	0
	Bond angle variance σ^2 (°^{2})	6.826	6.829	6.826	6.829	6.826	6.829	6.826	6.829	6.827	0.001
	Quadratic elongation	1.002	1.002	1.002	1.002	1.002	1.002	1.002	1.002	1.002	0
	Effective coordination number	5.994	5.994	5.994	5.994	5.994	5.994	5.994	5.994	5.994	0
$(NDA)(MA)Pb_2I_7$	Average Bond Length (Å)	3.214	3.214	3.214	3.214	3.214	3.214	3.214	3.214	3.214	0
	Polyhedral volume (Å^3)	44.044	44.045	44.044	44.045	44.046	44.045	44.046	44.045	44.045	0.001
	Distortion index (×10^{-2})	0.954	0.952	0.954	0.952	0.956	0.952	0.956	0.951	0.953	0.002
	Bond angle variance σ^2 (°^{2})	11.802	11.803	11.802	11.801	11.798	11.800	11.798	11.798	11.800	0.002
	Quadratic elongation	1.003	1.003	1.003	1.003	1.003	1.003	1.003	1.003	1.003	0
	Effective coordination number	5.974	5.974	5.974	5.974	5.974	5.974	5.974	5.974	5.974	0
$(PEA)_2(MA)Pb_2I_7$	Average Bond Length (Å)	3.196	3.197	3.196	3.197	3.196	3.197	3.196	3.197	3.197	0
	Polyhedral volume (Å^3)	43.332	43.307	43.332	43.307	43.332	43.307	43.332	43.307	43.320	0.013
	Distortion index (×10^{-2})	1.307	1.162	1.307	1.162	1.307	1.162	1.307	1.162	1.235	0.072
	Bond angle variance σ^2 (°^{2})	11.543	13.993	11.543	13.986	11.543	13.993	11.543	13.993	12.767	1.224
	Quadratic elongation	1.004	1.004	1.004	1.004	1.004	1.004	1.004	1.004	1.004	0
	Effective coordination number	5.933	5.953	5.933	5.953	5.933	5.953	5.933	5.953	5.943	0.01
$(DAH)(MA)Pb_2I_7$	Average Bond Length (Å)	3.21	3.213	3.21	3.213	3.21	3.213	3.21	3.213	3.212	0.001
	Polyhedral volume (Å^3)	43.548	43.61	43.549	43.608	43.549	43.609	43.549	43.608	43.579	0.030
	Distortion index (×10^{-2})	2.027	1.883	2.026	1.884	2.028	1.882	2.026	1.884	1.955	0.072
	Bond angle variance σ^2 (°^{2})	30.099	32.732	30.099	32.724	30.097	32.731	30.099	32.724	31.413	1.315
	Quadratic elongation	1.009	1.010	1.009	1.010	1.009	1.010	1.009	1.010	1.010	0
	Effective coordination number	5.831	5.885	5.831	5.885	5.831	5.885	5.831	5.885	5.858	0.027

Table S4. Structural metrics for each Pb-I octahedra including average bond length, polyhedral volume, distortion index (D), quadratic elongation, bond angle variance, effective coordination number in the bottom Pb-I octahedral layer (L2) for various bilayer 2D-PVKs.

Supplementary Reference:

[1] Chen, Z.; Li, Z.; Hopper, T. R.; Bakulin, A. A.; Yip, H. L., Materials, photophysics and device engineering of perovskite light-emitting diodes. *Rep Prog Phys* **2021**, *84* (4), 046401.

[2] Chen, Z.; Hoye, R. L. Z.; Yip, H.-L.; Fiuza-Maneiro, N.; López-Fernández, I.; Otero-Martínez, C.; Polavarapu, L.; Mondal, N.; Mirabelli, A.; Anaya, M.; Stranks, S. D.; Liu, H.; Shi, G.; Xiao, Z.; Kim, N.; Kim, Y.; Shin, B.; Shi, J.; Liu, M.; Zhang, Q.; Fan, Z.; Loy, J. C.; Zhao, L.; Rand, B. P.; Arfin, H.; Saikia, S.; Nag, A.; Zou, C.; Lin, L. Y.; Xiang, H.; Zeng, H.; Liu, D.; Su, S.-J.; Wang, C.; Zhong, H.; Xuan, T.-T.; Xie, R.-J.; Bao, C.; Gao, F.; Gao, X.; Qin, C.; Kim, Y.-H.; Beard, M. C., Roadmap on perovskite light-emitting diodes. *J. Phys. Photonics* **2024**, *6* (3), 032501.
